# Semisynthetic Isomers of Fucosylated Chondroitin Sulfate Polysaccharides with Fucosyl Branches at a Non-Natural Site

**DOI:** 10.1021/acs.biomac.1c01112

**Published:** 2021-11-14

**Authors:** Giulia Vessella, Roberta Marchetti, Angela Del Prete, Serena Traboni, Alfonso Iadonisi, Chiara Schiraldi, Alba Silipo, Emiliano Bedini

**Affiliations:** † Department of Chemical Sciences, University of Naples Federico II, Complesso Universitario Monte S.Angelo, via Cintia 4, I-80126 Napoli, Italy; § Department of Experimental Medicine, Section of Biotechnology, University of Campania “Luigi Vanvitelli”, via de Crecchio 7, I-80138 Napoli, Italy

## Abstract

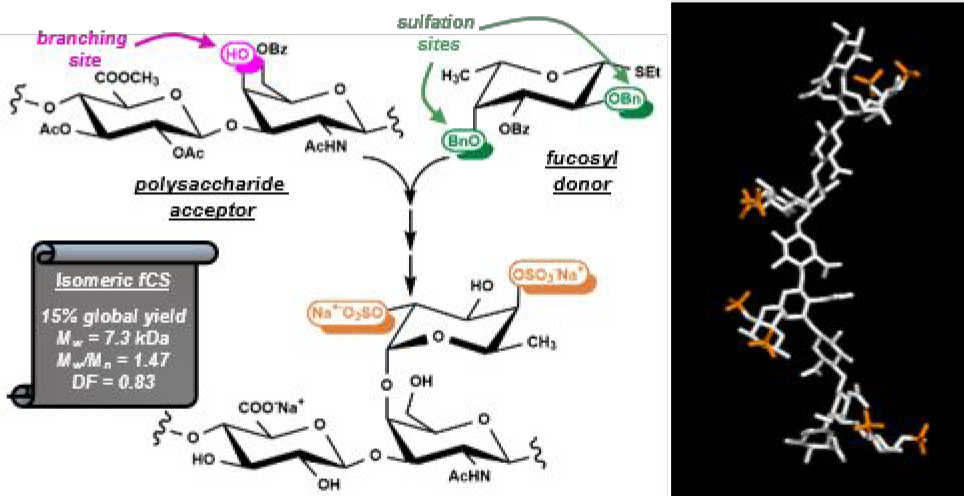

The several interesting activities detected for fucosylated chondroitin sulfate (fCS) have fueled in the last years several efforts toward the obtainment of fCS oligosaccharides and low molecular weight (LMW) polysaccharides with a well-defined structure, in order to avoid the problems associated with the potential employment of native, sea cucumber sourced fCSs as a drug. Total synthesis and controlled depolymerization of the natural fCS polysaccharides are the main approaches to this aim; nonetheless, they present some limitations. These could be circumvented by semisynthesis, a strategy relying upon the regioselective fucosylation and sulfation of a microbial sourced polysaccharide sharing the same chondroitin backbone of fCS but devoid of any fucose (Fuc) and sulfate decoration on it. This approach is highly versatile, as it could open access also to fCS isomers carrying Fuc and sulfate groups at non-natural sites. Here we prepare for the first time some structurally homogeneous fCS isomers through a multistep procedure with a glycosylation reaction between a LMW polysaccharide acceptor and three different Fuc donors as key step. The obtained products were subjected to a detailed structural characterization by 2D-NMR. The conformational behavior was also investigated by NMR and molecular dynamics simulation methods and compared with data reported for natural fCS.

## Introduction

Fucosylated chondroitin sulfate (fCS) is a glycosaminoglycan (GAG) found to date exclusively in the body wall of sea cucumbers (*Echinoidea*, *Holothuroidea*). It attracts constantly increasing interest for its activity in several biological events related to cellular growth, cancer metastasis, angiogenesis, inflammation, hyperglycemia, atherosclerosis, and, above all, coagulation and thrombosis.^1^ It is noteworthy that its anticoagulant and antithrombotic activity has been observed also on antithrombin (AT) and heparin cofactor II (HC-II)-free plasmas. This is due to some differences in the mechanism of action on the blood coagulation cascade^2^ with respect to unfractionated heparin, the most widespread and long-term used anticoagulant drug that is inactive on AT- and HC-II-free plasmas. In particular, fCS inhibits the intrinsic tenase and prothrombinase complexes, which are critical for activation of the blood coagulation cascade, as they generate factor Xa and thrombin.^3^ Furthermore, fCS retains its activity even with an oral administration, because it is digested neither in the gastric tract nor by intestinal bacterial enzymes.^4^ The possibility of an oral delivery and to exploit an alternative mechanism of actions are key aspects in the research for new anticoagulants drugs. For these reasons fCS stands as a promising anticoagulant drug candidate for heparin replacement.^5^ Indeed, although its long-standing and varied clinical applications, several studies indicated that heparin is not an ideal anticoagulant, especially for long-term therapies, due to many limitations such as induction of hemorrhagic side effects, thrombocytopenia, platelet release and aggregation, change in lipid metabolism, and osteoporosis.^6^

**Chart 1:**
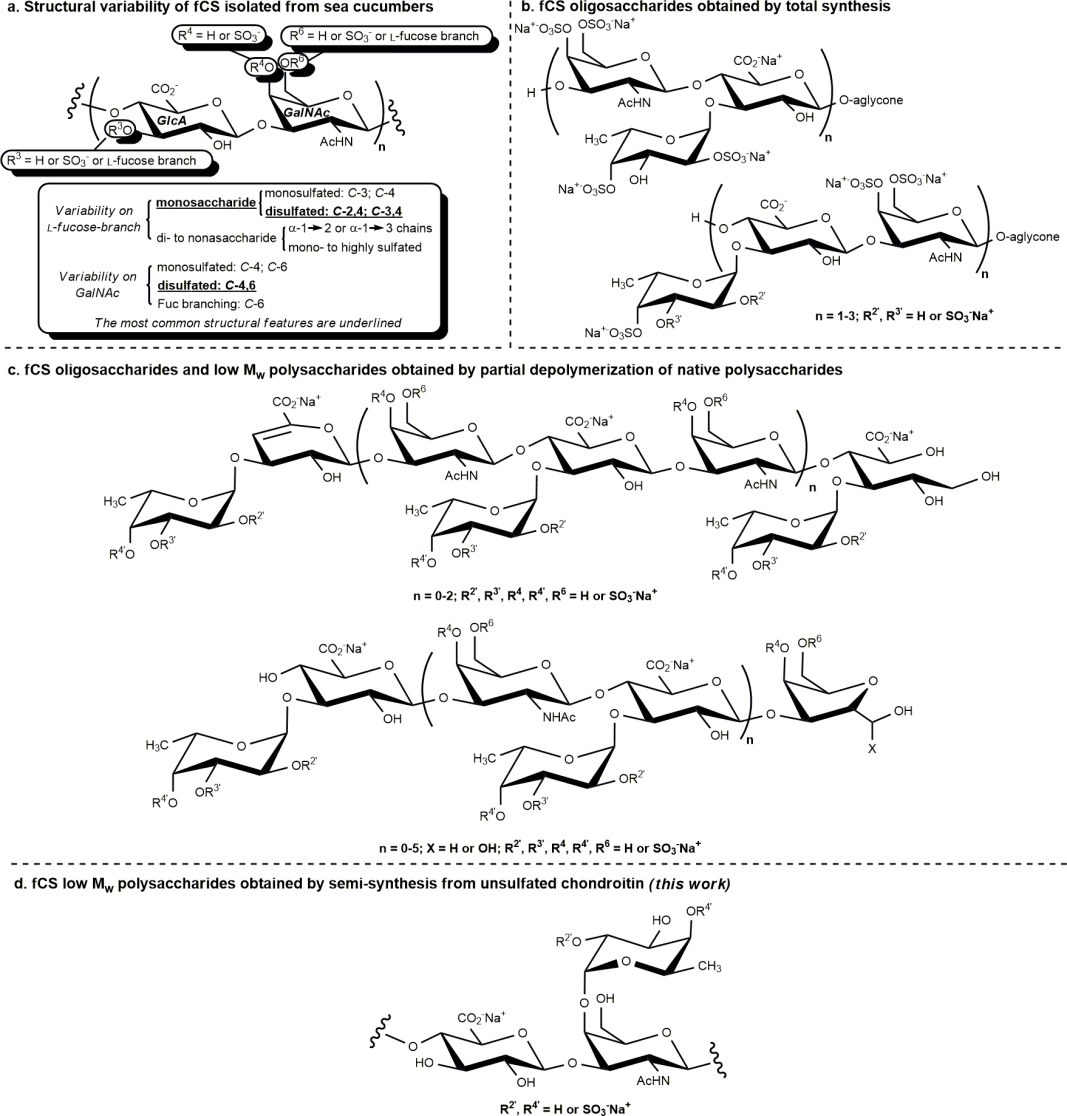
Structure of Natural fCS Polysaccharides and Chemically Obtained Oligosaccharides and Low *M*_w_ Polysaccharides

From a structural point of view, fCS shares the same backbone as chondroitin sulfate. It is composed of alternating 2-acetamido-2-deoxy-d-galactose (*N*-acetyl-galactosamine, GalNAc) and d-glucuronic acid (GlcA) units linked together through β-1–3 and β-1–4 glycosidic bonds, but with the unique peculiarity of variously sulfated 6-deoxy-l-galactose (l-fucose, Fuc) branches grafted on the polysaccharide chain, typically at *O*-3 position of GlcA units through an α-glycosidic bond (Chart 1a).^7^ Sulfate groups and Fuc branching are both essential to confer anticoagulant and antithrombotic activity to fCS, as demonstrated by the loss of activity shown by defucosylated and/or desulfated derivatives.^8^

Although fCS polysaccharides have shown very interesting biological activities, there are some ethical, ecological and biomedical concerns for their potential use as drug. Indeed, the sea cucumber source for fCS collides not only with the ever-stricter regulations for animal-derived drugs but also with the necessity to protect such species that offer key services to coastal sea ecosystems, buffering the effects of ocean acidification and facilitating the availability of nutrients and oxygen for other organisms in reef environments.^9^ Furthermore, fCS polysaccharides administration can cause some adverse effects, such as platelet aggregation, hypotension and bleeding, that are typically associated with high molecular weight (*M*_w_) sulfated species, as native fCS polysaccharides (their *M*_w_ typically spans from 25 to 140 kDa).^10^ To avoid this risk, in the past decade, several fCS oligosaccharides and low *M*_w_ polysaccharides have been produced^11^ through total synthesis^12−15^ or partial, selective depolymerization of natural fCS (Chart 1b,c).^10^ Even if the former approach can give access, at least theoretically, to whichever fCS species, including non-natural and multivalent architectures such as fCS glycoclusters,^16,17^ a very high number of chemical steps is typically required to obtain oligosaccharides not shorter than an octasaccharide, which seems to be the minimum structural unit able to confer anticoagulant activity.^18^ Conversely, the latter strategy allows the obtainment of the products in only one or few chemical steps, nonetheless their purification could be a difficult task if the employed depolymerization reaction is not selective enough and/or the native fCS is structurally heterogeneous. Actually, fCS polysaccharides very seldom display a homogeneous structure.^19−21^ Furthermore, even if some structural differences in terms of both sulfation and Fuc branching pattern could be found in dependence of the sea cucumber source,^7,9^ a limited number of fCS species can be accessed through the depolymerization approach (Chart 1c). Moreover, ethical and ecological problems discussed above are not solved at all.

To circumvent the limitations of the total synthetic and depolymerization approaches, an alternative strategy can rely upon the chemical modification of a microbial sourced exopolysaccharide (EPS) sharing the same backbone of fCS but lacking both sulfate groups and Fuc branches.^22^ Indeed, the number of steps for gaining fCS species is lowered with respect to total synthesis because most of glycosidic linkages are already present in the starting material, and at the same time a wide structural diversity is accessible provided that methods for the regioselective insertion of sulfate groups and Fuc branches on the EPS chain are available. This would allow the exploration of the chemical space around the native fCS structure. The achievement of this goal would furnish a highly valuable toolbox for expanding fCS structure–activity relationship (SAR) studies by investigating at a molecular level the role of Fuc branches in terms of both sulfate groups distribution and site of branching.

A controlled modification of polysaccharide structures is not a trivial task, due to several factors: (i) the difficulty in achieving regioselective derivatizations of the selected site(s) in each subunit of the polymer chain, (ii) the necessity to avoid harsh reaction conditions that might break the polysaccharide structure, and (iii) the generally poor solubility of many polysaccharides in commonly employed solvents. Although these limitations are particularly stringent in the case of GAG manipulation,^23^ semisynthetic routes to convert an unsulfated chondroitin EPS (CS-0) from *Escherichia coli* O5:K4:H4^24^ into low *M*_w_ fCS polysaccharides with different sulfation pattern and position of Fuc branches have been recently reported.^25−27^ Some of them exhibited a promising activity in preliminary anticoagulant assays, nonetheless, detailed SAR investigations were hampered by the structural heterogeneity of the obtained polysaccharides, that was in several cases even higher than that found in natural fCS species. In particular, the semisynthetic polysaccharides accessed up to now showed no regioselectivity and a too low degree of substitution (DS) for Fuc branches grafting. In this work a completely new route was investigated in order to obtain semisynthetic isomers of native fCS polysaccharides. They are characterized by a lower *M*_w_ and a higher structural homogeneity with respect to both natural and previously semisynthesized fCS species, carrying a high DS of Fuc units with a precise sulfation pattern and grafting position. Indeed, Fuc units are exclusively linked at GalNAc *O*-4 site (Chart 1d). To the best of our knowledge, up to now this branching position has been found in natural fCS polysaccharides only in a single case, but characterized by a very high structural heterogeneity.^28^

## Experimental Section

### General Methods

Commercial grade reagents and solvents were used without further purification, except where differently indicated. The term “pure water” refers to water purified by a Millipore Milli-Q gradient system. Centrifugations were performed at 4 °C (3500 g, 5 min) with an Eppendorf Centrifuge 5804 R instrument. Dialyses were conducted at 4 °C on Spectra/Por 3.5 kDa cutoff membranes. Freeze-dryings were performed with a 5Pascal Lio 5P 4K freeze-dryer. NMR spectra were recorded on a Bruker Avance III HD 400 MHz (^1^H NMR: 400 MHz, ^13^C NMR: 100 MHz) or on a Bruker Avance NEO (^1^H: 600 MHz, ^13^C: 150 MHz) instrument equipped with a cryo probe, in D_2_O (acetone as internal standard, ^1^H: (CH_3_)_2_CO at δ 2.22 ppm; ^13^C: (*C*H_3_)_2_CO at δ 31.5 ppm) or DMSO-*d*_6_ (^1^H: CHD_2_SOCD_3_ at δ 2.49 ppm; ^13^C: CD_3_SOCD_3_ at δ 39.5 ppm). Bruker TopSpin 4.0.5 software was used for all the experiments. Gradient-selected COSY and TOCSY experiments were performed using spectral widths of either 6000 Hz in both dimensions, using data sets of 2048 × 256 points. TOCSY mixing time was set to 120 ms. Transverse rotating-frame Overhauser enhancement spectroscopy (TROESY) and nuclear Overhauser enhancement spectroscopy (NOESY) experiments were performed using data sets (*t*_1_ × *t*_2_) of 2048 × 600 points with mixing times between 100 and 400 ms. ^1^H,^13^C-DEPT-HSQC experiments were measured in the ^1^H-detected mode via single quantum coherence with proton decoupling in the ^13^C domain, using data sets of 2048 × 256 points and typically 100 increments. PFG-NMR experiments were carried out at 298 K, by using a stimulated echo sequence with bipolar gradient pulses and one spoil gradient (stebpgp1s1d from Bruker library) with a longitudinal eddy current delay. Molecular mass analyses were performed by a Viscotek high-performance size-exclusion chromatographic (HP-SEC) system equipped with an integrated gel permeation chromatography system (GPCmax VE 2001, Viscotek, Malvern) and a triple detector array module (TDA 305, Viscotek, Malvern) including a refractive index detector (RI), a four-bridge viscosimeter (VIS), and a laser detector (LS) made of a right-angle light scattering (RALS) detector, and a low-angle light scattering (LALS) one. The employed analytical method was already extensively described.^29^

### Preparation of the Products

#### Derivative **2**

A fine suspension of polysaccharide **1**(27) (724 mg, 1.84 mmol repeating unit (RU)) in dry *N*,*N*-dimethylformamide (DMF, 34 mL) was stirred at 80 °C under an Ar atmosphere. After 2 h, the mixture was cooled to rt, treated with α,α,4-trimethoxytoluene (3.14 mL, 18.4 mmol), which was freshly dried over 4 Å MS, and then with a 0.24 M solution of (+)-camphor-10-sulfonic acid (CSA) in dry DMF (1.92 mL, 460 μmol). After 18 h of stirring at 80 °C under an Ar atmosphere, the obtained solution was cooled to rt and treated with cold acetone (70 mL). The obtained precipitate was collected by centrifugation and dried under vacuum overnight to give **2** (1.06 g, 146% mass yield) as a yellowish oil.

#### Derivative **3**

A suspension of **2** (949 mg, 1.90 mmol RU) in CH_3_CN (20 mL) was treated with triethylamine (Et_3_N, 5.71 mL, 38.0 mmol), acetic anhydride (Ac_2_O, 12.6 mL, 133 mmol), and 4-dimethylaminopyridine (DMAP, 115 mg, 941 μmol). After 19 h of stirring at rt, the obtained brown solution was treated with diisopropyl ether (280 mL) to give a precipitate that was collected by centrifugation and dried under vacuum overnight. Derivative **3** (1.35 g, 142% mass yield) was obtained as a yellowish oil.

#### Derivative **4**

A solution of **3** (1.32 g, 2.26 mmol RU) in 9:1 *v*/*v* acetic acid (AcOH)–H_2_O (5 mL) was stirred at 50 °C for 7 h, then cooled to rt and dialyzed. After freeze-drying, derivative **4** (670 mg, 51% mass yield) was obtained as a white powder.

#### Derivatives **5-a–f**

A solution of polysaccharide **4** (39.8 mg, 83.3 μmol RU) in pyridine (1.7 mL) was treated with benzoyl cyanide (BzCN, 109 mg, 831 μmol for **5-a,b**; 54.6 mg, 418 μmol for **5-c**; 27.3 mg, 208 μmol for **5-d**) or pivaloyl chloride (PivCl, 50.4 mg, 418 μmol for **5-e,f**). After stirring at rt (for **5-a,c–e**; or 4 °C for **5-b,f**) for 4 h (for **5-b–f**; or 24 h for **5-a**), the reaction was quenched by addition of CH_3_OH (700 μL). The mixture was then treated with cold diisopropyl ether (10 mL). The obtained precipitate was collected by centrifugation and then dried under vacuum overnight to afford derivatives **5-a–f** (47.4 mg, 119% mass yield for **5-a**; 37.6 mg, 95% mass yield for **5-b**; 47.2 mg, 119% mass yield for **5-c**; 39.1 mg, 98% mass yield for **5-d**; 80.9 mg, 203% mass yield for **5-e**; 87.1 mg, 219% mass yield for **5-f**) as white powders.

#### Derivatives **6-a–f**

A solution of **5-a** (34.1 mg, 58.6 μmol RU) in dry DMF (700 μL), was treated with a 0.71 M solution of pyridine–sulfur trioxide complex (SO_3_·py) in dry DMF (1.5 mL) and then stirred overnight at 50 °C. A cold, saturated NaCl solution in acetone (10 mL) was added to give a yellowish precipitate that was collected by centrifugation and then suspended in deionized water (2.0 mL). The resulting acid solution (pH ∼ 2) was heated to 50 °C and stirred for 2 h. Then, a 4 M NaOH solution in water was added to adjust pH to 12. The solution was stirred at rt overnight and then neutralized by dropwise addition of 1 M HCl. Dialysis and subsequent freeze-drying gave polysaccharide **6-a** (20.3 mg, 60% mass yield for **6-a**; 17.2 mg, 50% mass yield for **6-b**; 23.2 mg, 68% mass yield for **6-c**; 19.7 mg, 58% mass yield for **6-d**; 5.4 mg, 16% mass yield for **6-e**; 10.2 mg, 30% mass yield for **6-f**) as a white amorphous solid.

#### Derivatives **8-a–c**

A mixture of polysaccharide acceptor **5-c** (64.0 mg, 110 μmol RU) and fucosyl donor **7-a** (132 mg, 275 μmol; or 139 mg, 275 μmol for **7-b**; or 136 mg, 275 μmol for **7-c**) was coevaporated three times with dry toluene (3 mL each). The residue was dried under vacuum and then mixed, under an Ar atmosphere, with AW-300; 4 Å-MS. DMF (3.9 mL) and CH_2_Cl_2_ (6.5 mL), which were freshly dried over AW-300 4 Å-MS, were added to the solid mixture. The mixture was stirred at rt for 10 min, and then treated under Ar atmosphere with *N*-iodosuccinimide (NIS, 68.1 mg, 303 μmol) and a 0.62 M solution of trimethylsilyl trifluoromethanesulfonate (TMSOTf) in freshly dried CH_2_Cl_2_ (136 μL, 84.3 μmol). After 4 h stirring at rt, a few drops of Et_3_N were added to quench the reaction. Then, the molecular sieves were removed by decantation and the mixture was poured into diisopropyl ether (30 mL). The obtained precipitate was collected by centrifugation and dried under vacuum overnight. The product was then subjected to a second glycosylation step, reiterating the same procedure, affording **8-a** (88.2 mg, 138% mass yield; or 83.2 mg, 130% mass yield for **8-b**; or 86.3 mg, 135% mass yield for **8-c**) as a yellowish powder.

#### Derivatives **9-a–c**

Derivative **8-a** (84.6 mg, 145 μmol RU; or 83.2 mg, 145 μmol RU for **8-b**; or 95.5 mg, 164 μmol RU for **8-c**) was dissolved in CH_3_CN (1.5 mL) and then treated with Et_3_N (405 μL, 2.91 mmol), Ac_2_O (960 μL, 10.2 mmol), and DMAP (7.1 mg, 58 μmol). After 21 h of stirring at rt, cold diisopropyl ether (7 mL) was added to give a precipitate that was collected by centrifugation, dried under vacuum overnight, and then suspended in ethyl acetate (2.6 mL). The mixture was treated with a 0.34 M solution of NaBrO_3_ in pure water (2.35 mL, 799 μmol). A 0.27 M solution of Na_2_S_2_O_4_ in pure water (2.40 mL, 648 μmol) was added portionwise over a period of 10 min. The triphasic mixture was vigorously stirred at rt overnight under visible light irradiation by warm LED lights. A precipitate was collected by centrifugation and dried under vacuum overnight affording **9-a** (53.0 mg, 63% mass yield; or 83.2 mg, 130% mass yield for **9-b**; or 35.3 mg, 37% mass yield for **9-c**) as a slightly yellow powder.

#### fCS Polysaccharides **10-a–c**

A solution of **9-a** (50.5 mg, 69.4 μmol RU; or 83.2 mg, 145 μmol RU for **9-b**; or 50.5 mg, 60.7 μmol RU for **9-c**) in dry DMF (1.5 mL) was treated with a 1.0 M solution of SO_3_·py in dry DMF (1.39 mL, 1.39 mmol) and then stirred at 50 °C for 17 h. The solution was then cooled to rt, and a saturated NaCl solution in acetone (18 mL) was added to give a precipitate that was collected by centrifugation and then suspended in pure water (1.5 mL). The suspension was treated with a 4 M NaOH solution in water to adjust the pH to 12. After 6 h of stirring at rt, the obtained solution was neutralized by adding a 4 M HCl solution in water. Dialysis and subsequent freeze-drying gave a slightly yellow powder that was further purified by filtration on a Sep-Pak C18 cartridge followed again by freeze-drying. Finally, fCS polysaccharide **10-a** (8.1 mg, 16% mass yield; or 83.2 mg, 130% mass yield for **10-b**; or 15.4 mg, 30% mass yield for **10-c**) was obtained as a white waxy solid.

#### Molecular Dynamic Simulations

fCS three-dimensional structures were built with the Carbohydrate Builder of GLYCAM web site (http://glycam.org/). The generated structures were prepared for molecular dynamics (MD) simulation using the tLEaP module of AMBER18; GLYCAM06j-1 force field was employed to represent the carbohydrate parameters.^30^ Prior to MD simulations, counterions were added to neutralize the system, and then it was solvated with an octahedral box of explicit TIP3P water, extending 15 Å away from any atom. The MD simulations were performed using the CUDA^31,32^ implementation of PMEMD in the AMBER18 software.^33^ Minimization was performed using Sander and MD simulations were performed using the CUDA^31,32^ implementation of PMEMD in the AMBER18 software. The MD simulations were carried out under periodic boundary conditions using the smooth particle mesh Ewald method to represent the systems’ long-range electrostatic interactions. For the equilibration phase, initial annealing of the system was brought from 100 to 300 K over 25 ps. The temperature was kept constant at 300 K during 50 ps with progressive energy minimizations and a solute restraint. The restraints were gradually released by the solute, which was closely followed by a 20 ps heating period that went from 100 to 300 K; once completed, the restraints were removed. Last, a production simulation of 100 ns was carried out in an isothermal–isobaric ensemble. Coordinates were collected to acquire 10000 structures of the progression of the dynamics. Trajectories were processed and analyzed with the cpptraj module included in the AmberTools suite software package and visualized with the VMD molecular visualization program.^34^

## Results and Discussion

**Scheme 1:**
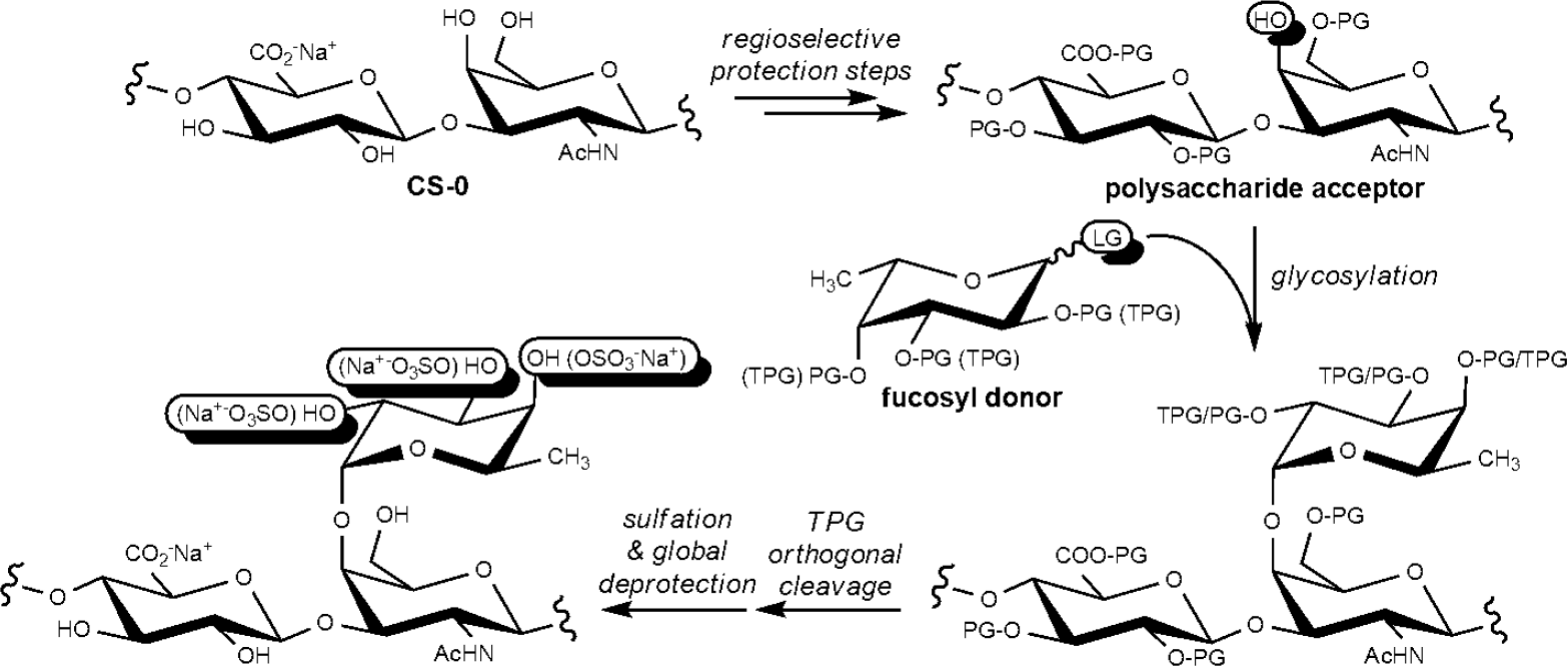
General Strategy to Access Semisynthetic Isomers of fCS Polysaccharides from Microbial-Sourced Unsulfated Chondroitin PG = protecting group; TPG = temporary protecting group; LG = leaving group.

**Scheme 2:**
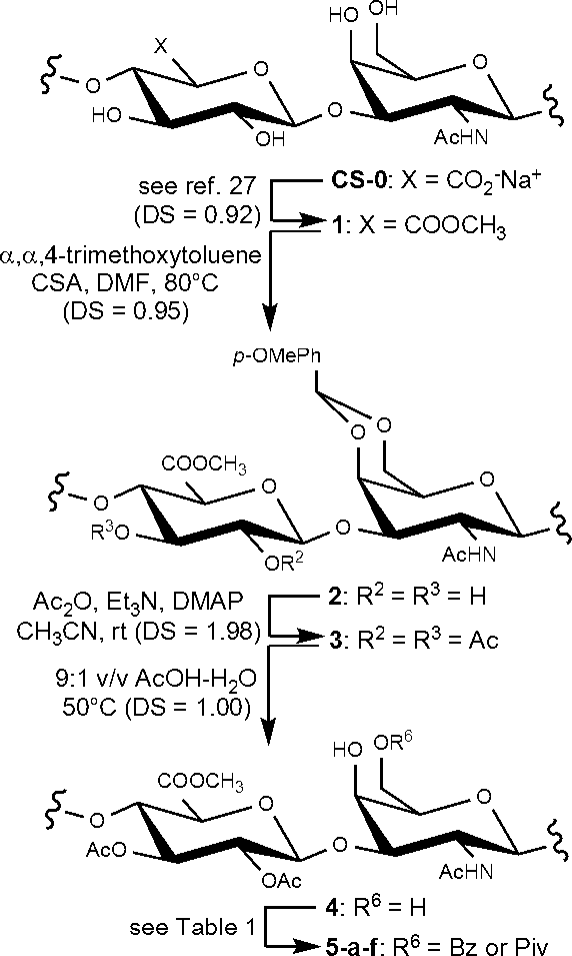
Semisynthesis of a Polysaccharide Acceptor with a Single Free Hydroxyl at the GalNAc *C*-4 Position

The general strategy to obtain semisynthetic, homogeneously grafted, and sulfated fCS polysaccharides starting from microbial-sourced CS-0 is to convert the latter into a derivative with every position of the disaccharide repeating units protected except one, through a suitably optimized multistep procedure. Such derivative, termed polysaccharide acceptor, can be then branched with Fuc units by a glycosylation reaction employing suitably protected fucosyl donors. Further structural manipulations, including an orthogonal cleavage of temporary protecting groups located at positions to be sulfated, followed by sulfation and global deprotection, can furnish the target semisynthetic fCSs (Scheme 1). Since the latter were designed with Fuc units selectively linked at GalNAc *O*-4 positions, the first aim of the work was the semisynthesis of a polysaccharide acceptor with a single free hydroxyl at such a site on every disaccharide repeating unit of the polymer. To reach this goal, known polysaccharide **4**(25) was first obtained through a modified procedure. Microbial-sourced CS-0 was converted into its methyl ester derivative **1**(27) that was subjected in turn to the installation of a *p*-methoxybenzylidene protecting group on GalNAc 4,6-diol employing α,α,4-trimethoxytoluene with CSA as catalyst in DMF (Scheme 2). Degree of substitution (DS) for the *p*-methoxybenzylidene group was evaluated equal to 0.95 from the relative integration of the acetal CH (δ 5.46 ppm) and *N*-acetyl (δ 1.77 ppm) signals in the ^1^H NMR spectrum of **2** (Figure S1). To the best of our knowledge, this was the first example of a *p*-methoxybenzylidene acetal protection on a polysaccharide structure with an almost quantitative DS.

After acetylation of GlcA 2,3-diol, the obtained derivative **3** (DS = 1.98, as calculated by ^1^H NMR integration, Figure S2) was subjected to selective hydrolysis of the *p*-methoxybenzylidene acetal under conditions mild enough to avoid breakage of the glycosidic bonds of the polysaccharide. Noteworthy, with respect to an unsubstituted benzylidene acetal, *p*-methoxybenzylidene one is more labile to acid hydrolysis. This allowed a shortening of reaction time (from 48 to 7 hours) for the quantitative conversion of fully protected derivative **3** to diol **4** with respect to the same reaction conducted on the benzylidene-protected counterpart of **3**.^25^ In order to access a polysaccharide acceptor with a single hydroxyl free at the GalNAc *C*-4 position, the regioselective acylation of the primary hydroxyl of the 4,6-diol in **4** was investigated. The acylating agents benzoyl cyanide (BzCN) and pivaloyl chloride (PivCl) were selected because they have been already employed for the selective protection of primary versus secondary alcohol moieties on short chain chondroitin oligosaccharides^35^ and on other polysaccharides.^36^ Several, different reaction conditions for the acylation steps were tested, changing temperature, reaction time, and acylating agent equivalents (Table 1). DS and regioselectivity of the reactions could not be easily evidenced from ^1^H NMR spectra of the protected products due to the presence of acylating reagent impurities overlapping the polysaccharide signals to be integrated (Figures S4 and S5). Therefore, products **5-a–f**, collected by precipitation from the acylation crude mixture, were directly subjected to sulfation under standard conditions with the pyridine–sulfur trioxide (SO_3_·py) complex in DMF, in order to mark the positions carrying a free hydroxyl in **5-a–f**. After a global deprotection under alkaline hydrolytic conditions, semisynthetic CS polysaccharides **6-a–f** were obtained.

**Table 1:**
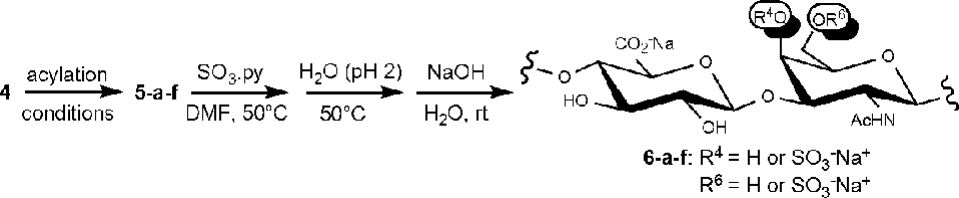
Optimization of Reaction Conditions for Regioselective Acylation of Derivative **4** and Semisynthesis of CS Polysaccharides **6-a–f**

CS product	acylation conditions	yield^a^ (%)	4S/4-OH ratio^b^	6S/6-OH ratio^c^
**6-a**	BzCN (10 equivs)	75	55:45	n.d.^d^
	py, rt, 24 h			
**6-b**	BzCN (10 equivs)	50	53:47	n.d.^d^
	py, 4 °C, 4 h			
**6-c**	BzCN (5 equivs)	86	82:18	n.d.^d^
	py, rt, 4 h			
**6-d**	BzCN (2.5 equivs)	60	90:10	13:87
	py, rt, 4 h			
**6-e**	PivCl (5 equivs)	34	86:14	n.d.^d^
	py, rt, 4 h			
**6-f**	PivCl (5 equivs)	69	87:13	29:71
	py, 4 °C, 4 h			

a Overall mass yield determined over 7 steps from **1**.

b Estimated as the percentage ratio between the integral of GalNAc4S CH-4 peak volume and the integral of GalNAc0S CH-4 peak volume in the ^1^H,^13^C-DEPT-HSQC spectrum (Figures S6–S11).

c Estimated as the percentage ratio between the integral of GalNAc6S CH_2_-6 peak volume and the integral of GalNAc0S and GalNAc4S CH_2_-6 peak volume in the ^1^H,^13^C-DEPT-HSQC spectrum (Figures S6–S11).

d Not detected.

**Figure 1:**
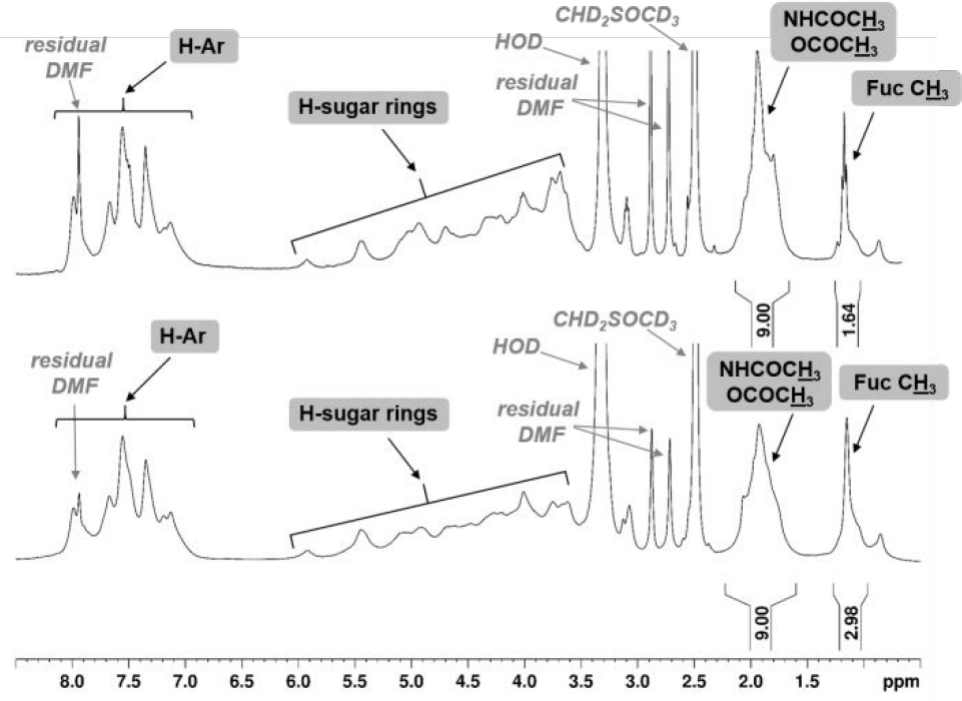
^1^H NMR spectra (600 MHz, DMSO-*d*_6_, 298 K) of **8-b** after one (up) or two (down) fucosylation steps. Polysaccharide signal assignments are enclosed in rectangles.

**Scheme 3:**
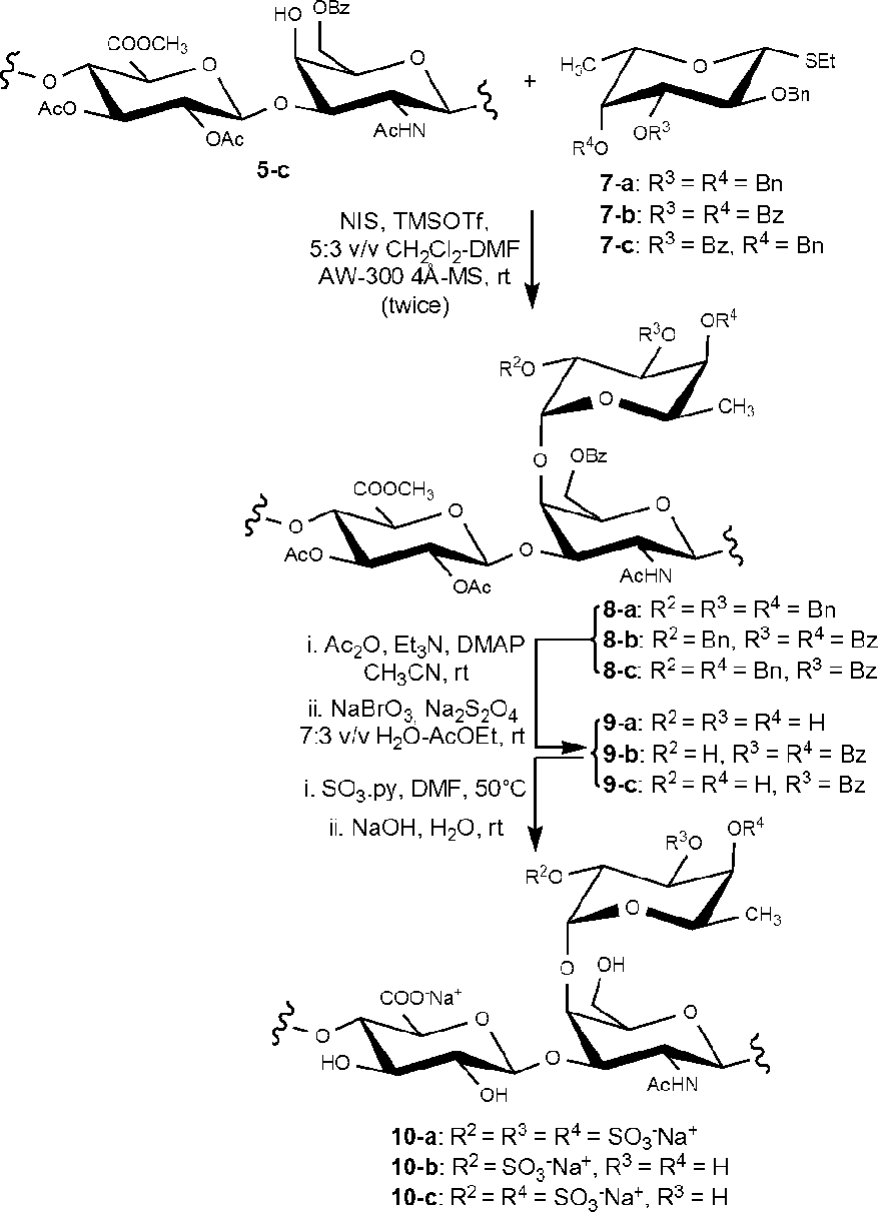
Semisynthesis of fCS Polysaccharides **10-a–c** Postulated structures are depicted (for structural characterization, see text and Table 2).

All the obtained CS polysaccharides were subjected to a detailed structural characterization through 2D-NMR spectroscopy and comparison of the extracted chemical shifts with literature data.^37^ In particular, two signals attributable to GalNAc CH-4 atoms of 4-sulfated or 4-unsulfated units could be detected in DEPT-HSQC spectra (Figures S6–S11) at δ_H/C_ 4.72/77.6 and 4.09/68.9 ppm, respectively. The relative integration of their volumes was possible assuming that the signals displayed similar ^1^*J*_C,H_ coupling constants and that a difference of around 5–8 Hz from the experimental set value did not cause a substantial variation of the integrated peak volumes.^38^ It resulted in a nearly equivalent percentage of GalNAc4S and GalNAc0S units in **6-a** and **6-b** (Table 1, entries 1,2), thus, suggesting a protection as Bz esters of some of GalNAc 4-OHs of the polysaccharide, together with all GalNAc 6-OHs, during the conversion of **4** into **5-a,b** with 10 equivs BzCN. By lowering the amount of acylation reagent to 5 equivs, a markedly enhanced regioselectivity could be gained in both the benzoylation and the pivaloylation reaction, as evidenced by the higher GalNAc4S/GalNAc0S ratio in the related final products **6-c,e** (entries 3 and 5). A decrease of reaction temperature or a further reduction of the acylation reagent had to be discarded, as a non-negligible signal at δ_H/C_ 4.24/68.9 ppm, attributable to CH_2_ atoms of 6-sulfated GalNAc residues,^37^ appeared in the DEPT-HSQC spectra (Figures S9–S11) of **6-d,f**. Most likely, this was due to a nonquantitative protection of the primary alcohols at the GalNAc 6-site under the mildest acylation conditions giving **5-d,f**. Therefore, **5-c,e** were revealed to be the most regioselectively acylated derivatives. However, **5-c** was preferred as the polysaccharide acceptor in subsequent fucosylation reactions, because the lower hindrance of Bz with respect to Piv protecting groups would be expected to be a favorable steric factor conferring a higher nucleophilicity to the adjacent hydroxyl on the GalNAc *C*-4 in the fucosylation reactions. The glycosylation was performed with fucosyl donors **7-a–c**,^26,39^ having a different pattern of temporary (Bn ether) and permanent (Bz ester) protecting groups (Scheme 3), under activation of the thioglycoside leaving group with NIS and TMSOTf in a DMF–CH_2_Cl_2_ solvent mixture ensuring a good α-stereoselectivity.^40^ DS for fucosylations conducted on polysaccharide derivatives other than **5-c** and a similar set of Fuc donors shifted from moderate to good values when performed twice, but no further DS increase was observed when it was repeated thrice (data not shown). Therefore, glycosylation reactions of **5-c** were performed twice with 2.5 equiv of Fuc donor **7-a–c** per repeating unit for each glycosylation. Branched polysaccharide derivatives **8-a–c** were recovered by precipitation from the crude glycosylation mixture and subjected to ^1^H NMR analysis. A DS estimation could be obtained by relative integration of *N*,*O*-acetyl and Fuc methyl signals at δ 2.1–1.7 and 1.2–1.1 ppm, respectively. Although Fuc signal and, therefore, DS could be overestimated if some byproducts coming from Fuc donor coprecipitate with the polysaccharide, a DS enhancement (estimated from 0.55 to 0.99 for **8-b**) could be clearly detected from the comparison of the ^1^H NMR spectra obtained after a single or two fucosylation reactions (Figure 1).

Hence, **8-a–c** were subjected to the rest of the multistep sequence to gain semisynthetic fCS polysaccharides **10-a–c** (Scheme 3). In particular, capping of any unreacted hydroxyl was first conducted by acetylation, followed by cleavage of the temporary Bn ether protecting groups under oxidative conditions.^41^ The restored alcohol moieties of **9-a–c** were then sulfated and finally a global deprotection of ester protecting groups by alkaline hydrolysis completed the semisynthetic sequence.

The semisynthetic fCS species **10-a–c**, obtained in 14–25% overall mass yield from acceptor **5-c**, were first subjected to an evaluation of their weight-averaged *M*_w_ by high-performance size exclusion chromatography combined with a triple detector array (HP-SEC-TDA).^42,43^ A much lower *M*_w_ was detected for **10-a–c** (6.4–8.7 kDa, Table 2) with respect to natural fCS polysaccharides (25–140 kDa),^10^ as expected from the starting *M*_w_ of microbial-sourced CS-0 (23.7 kDa)^27^ that was further lowered presumably in the acid-mediated reactions (*p*-methoxybenzylidene insertion and cleavage, fucosylation, Bn ether oxidative deprotection and sulfation) of the semisynthetic sequence, but without a substantial increase of the polydispersity (*M*_w_/*M*_n_) range (1.22–1.47 for **10-a–c**; 1.34 for starting CS-0). Noteworthy, fCS species with a *M*_w_ of 8–12 kDa are known to retain the activity of the native polysaccharides, while minimizing the undesired effects (i.e., bleeding, platelet aggregation), exhibited by the latter.^44−46^

**Table 2:** Yield and Structural Data of fCS Polysaccharides **10-a–c**

product	yield^a^ (%)	*M*_w_^b^ (kDa)	*M*_w_/*M*_n_^c^	DF^d^
**10-a**	14	8.7	1.22	0.66
**10-b**	25	6.4	1.26	0.71
**10-c**	15	7.3	1.47	0.83

a Overall mass yield determined over 5 steps from **5-c**.

b *M*_w_ = weight-averaged molecular weight.

c *M*_n_ = number-averaged molecular weight.

d Determined by ^1^H NMR integration of Fuc methyl and GalNAc *N*-acetyl signals.

**Figure 2:**
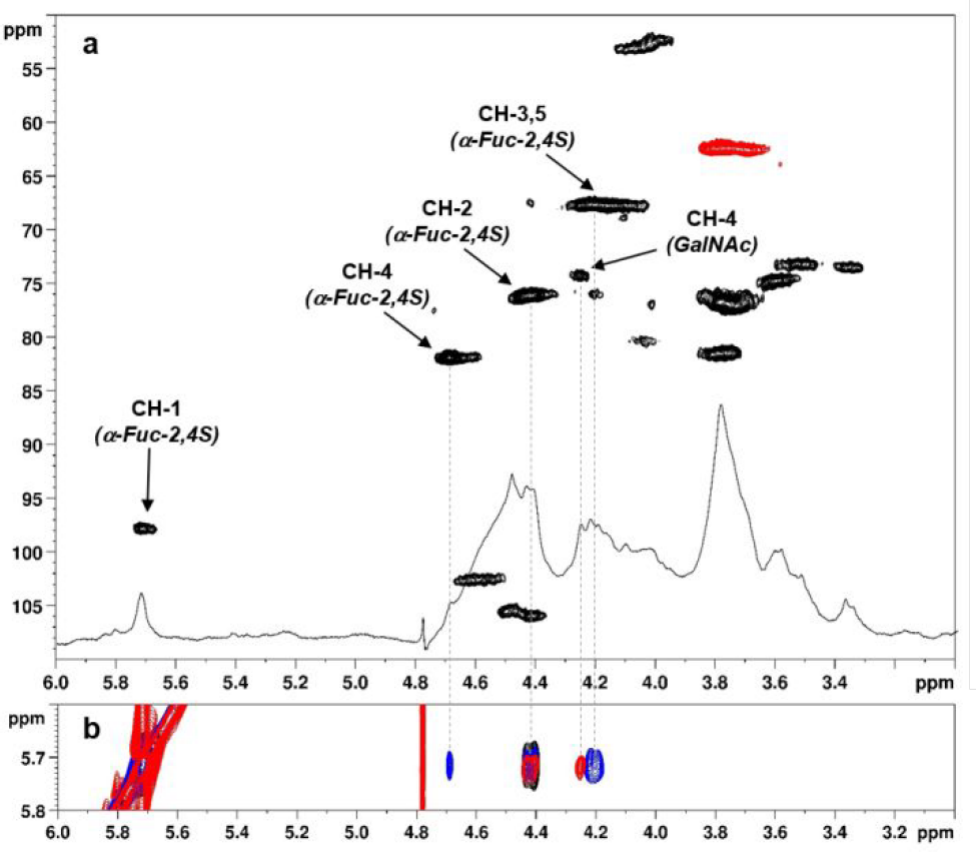
(a) ^1^H and ^1^H,^13^C-DEPT-HSQC and (b) COSY (black), TOCSY (blue), and NOESY (red) NMR spectra (600 MHz, D_2_O, 298 K, zoom) of **10-c**. Only some of the assignments are indicated (for full assignments, see the Supporting Information).

The postulated sulfation and fucosylation patterns for **10-a–c** were scrutinized by ^1^H- and 2D-NMR analysis. Polysaccharide **10-c** was analyzed first. A ^1^H DOSY NMR spectrum (Figure S13) confirmed the covalent attachment of Fuc to chondroitin backbone. The ^1^H,^13^C-DEPT-HSQC spectrum (Figure 2a) clearly showed the presence of a single signal at ^1^H-chemical shift over 5 ppm (δ_H/C_ 5.71/98.0 ppm), easily assigned to α-configured anomeric Fuc CH atoms.^47^

Cross-peaks related to this signal in COSY and TOCSY spectra (Figure 2b) allowed the assignment of the chemical shifts for Fuc ring CH atoms. The downfield shift detected for CH-2 and CH-4 (δ_H/C_ 4.43/76.4 and 4.69/82.1 ppm, respectively) with respect to CH-3 (δ_H/C_ 4.19/67.8 ppm) confirmed the postulated 2,4-sulfation pattern for α-Fuc units (Scheme 3). The analysis of the cross peaks in the NOESY spectrum showed a correlation of anomeric α-Fuc signal with a density at δ_H_ 4.26 ppm, attributable to GalNAc H-4 by means of the other 2D-NMR spectra, thus, confirming the expected position for Fuc branching and ruling out the hypothetical Fuc attachment at nitrogen atom of GalNAc to afford an imidate moiety, as reported for glycosylations on GlcNAc-containing oligosaccharide acceptors.^48^

An evaluation of the degree of fucosylation (DF) could be obtained by relative integration of Fuc methyl and GalNAc *N*-acetyl signals at δ_H_ 1.36–1.21 and 2.05–1.94 ppm, respectively, in the ^1^H NMR spectrum. The obtained 0.83 value (Table 2) overtakes all the DFs reported up to now for semisynthetic fCS.^25−27^ The absence of any distinguishable signal in 2D-NMR spectra related to regio- or stereoisomerically linked Fuc other than α-1 → 4-units as well as to Fuc sulfation patterns, other than the 2,4-one, accounted for a rather high degree of structural homogeneity, at least comparable with that found in natural fCS polysaccharides and surely much higher with respect to semisynthetic fCSs accessed up to now.^25−27^ DF, Fuc branching site, and sulfation pattern as well as a full NMR signal assignment could be similarly evaluated for differently sulfated fCS polysaccharides **10-a,b** (Figures S15 and S16 and Table S1). In the case of Fuc2S-branched derivative **10-b**, the postulated structure could be confirmed, whereas to our surprise, no sulfate groups could be found in **10-a**, for which a trisulfated Fuc2,3,4S-branched structure was postulated instead. The chemical shifts values for Fuc-2,3,4 CH atoms (δ_H/C_ 3.76/69.8, 3.92/70.9, and 3.81/73.1 ppm, respectively; see Table S1) were indicative for the presence of unsulfated Fuc branches in **10-a**.^49^^1^H NMR spectrum of its precursor **9-a** in the semisynthetic sequence showed the absence of an aromatic signal at δ 7.15–7.35 ppm typical for benzyl moieties (Figure S12), thus, ruling out the hypothesis of a failure in the oxidative removal of Bn ether protecting groups on Fuc units. Alternatively, this surprising recalcitrance of Fuc-2,3,4-triol to be sulfated could be hypothetically due to a strong hydrogen bond network between inter-residue alcohol moieties in **9-a**, hindering any solvent and reagent approach in a much higher measure than what isolated hydroxyls can do in **9-b** and **9-c** (precursors to 2,4- and 2-sulfated fCS products **10-b** and **10-c**, respectively), as well as what 2,3,4-triol moieties can do in randomly distributed^26,27^ rather than in regioselectively branching Fuc units.

**Figure 3:**
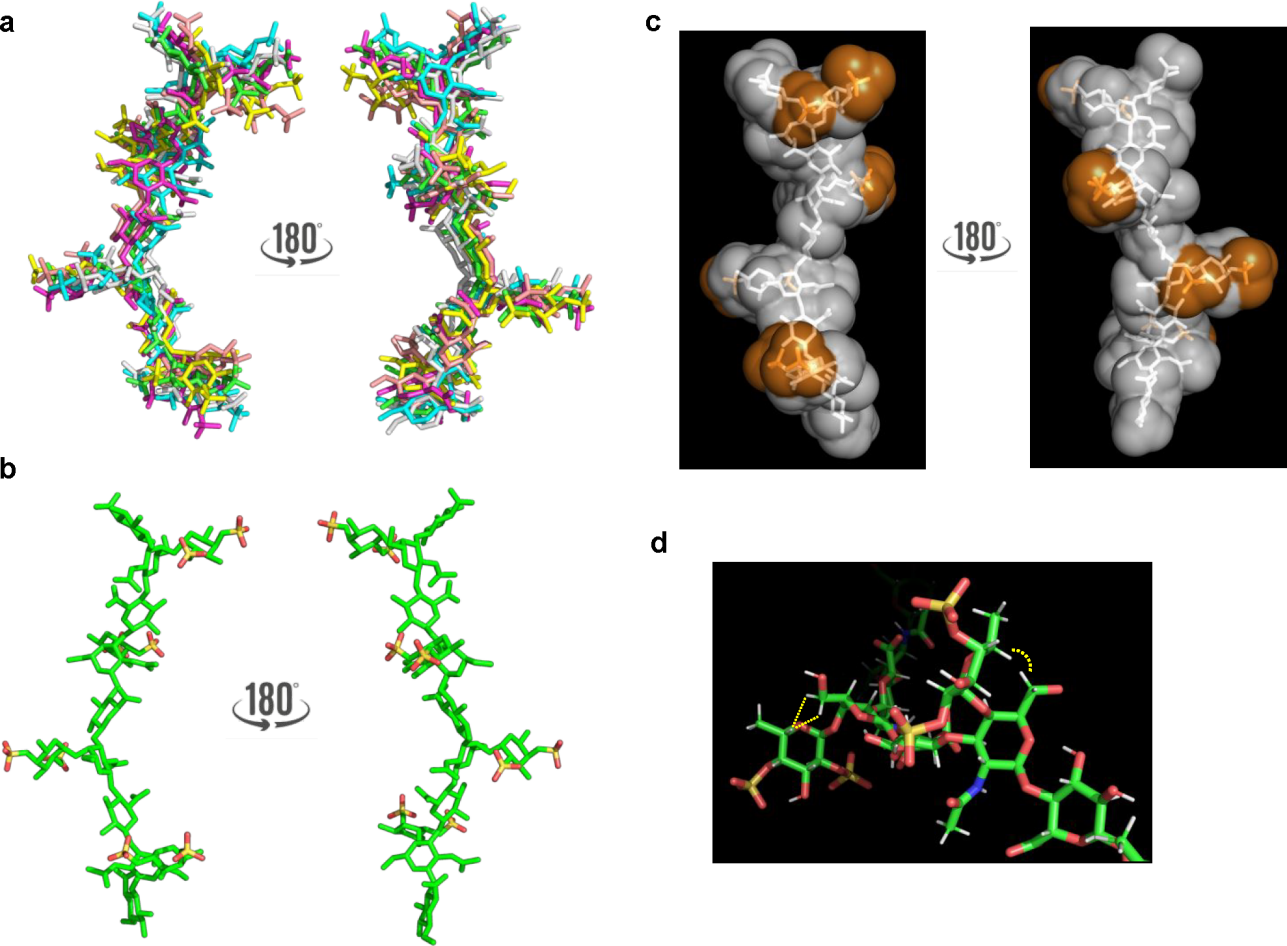
(a) Representative structures of the most populated clusters of fCS **10-c** extracted from the MD simulation (each structure is differently colored). (b, c) Structure and molecular surface of fCS **10-c** structure of a representative conformer of the most populated cluster from the MD simulation (fCS backbone is represented in white and sulfate groups are represented in orange). (d) Key distances between GlcNAc H-6 and Fuc H-5 (diagnostic of the orientation of the Fuc ring with respect to the GalNAc-GlcA skeleton).

In order to compare the conformational behavior of the here obtained fCS isomers with their natural counterparts, a 3D modeling study was accomplished through NMR and molecular dynamics (MD) simulation techniques. A similar investigation has been reported on a disulfated Fuc-branched fCS from sea cucumber *Holothuria forskali*.^50^ Therefore, for an optimal comparison, the conformational behavior of semisynthetic fCS **10-c**, displaying disulfated Fuc branches as well, was investigated by molecular mechanics and dynamic simulations and NOE-based NMR experiments. The potential energy surfaces of the three disaccharides constituting fCS **10-c** repeating unit were constructed and the energetically accessible conformational regions were evaluated. The corresponding adiabatic energy maps for the glycosidic torsions Φ (H1–C1–O–CX′) and Ψ (C1–O–CX′–HX′) showed global minima in accordance with the *exo*-anomeric effect (Figure S18). A moderate flexibility around Φ torsion and higher flexibility around Ψ angle was detected, corresponding to *exo*-Φ/*syn*-Ψ conformations. To gain further conformational insights, the behavior of fCS **10-c** oligosaccharides embedding one repeating unit and four repeating units was investigated by MD simulation using AMBER18. Starting from Φ and Ψ minima obtained by MM calculation, a dodecasaccharide encompassing four repeating units was constructed, and the conformational space available was next investigated by MD simulations. The initial structures were extensively minimized and subjected to a MD simulation of 100 ns in explicit water with AMBER18. Cluster and dihedral analysis were performed and showed that the dodecasaccharide was stable for most of the simulation in an extended topology (>95% of the MD time, Figure 3). The corresponding Φ/Ψ scatter plots, displayed in Figure S19, confirmed the conformational regions energetically accessible to the disaccharide units and the preference for the *exo*-anomeric conformation around all the glycosidic linkages. For all the MD simulations, ensemble average interproton distances were extracted and translated into NOE contacts according to a full-matrix relaxation approach. Notably, the average distances obtained for the MD simulation from ⟨r^–6^⟩ values were compared to those collected experimentally, and an excellent accordance between the experimental and calculated data was found. The dihedral angle populations of GlcA-1 → 3-GalNAc and GalNAc-1 → 4-GlcA linkages were conserved across the units and were consistent with the structures of other CS oligosaccharides (Figure S19).^51−53^ It has been demonstrated how the conformation of fCS is characterized by a tight arrangement of the trisaccharide repeating units very similarly to Le_*x*_ blood antigen trisaccharide conformation, and how a high level of sulfation does not alter this conformation, with the Fuc residue linked at position 3 of GalA stacking on top of the GalNAc unit.^50^ Here, Fuc is linked to the GalNAc unit; the stacking with this residue was not observed, while the NOE contact of Fuc H-5 and (very low) H-3 with GalNAc H-6 (Figure S14) suggested an orientation of Fuc ring almost perpendicular to the GalNAc and GlcA units (Figures 3 and S20).

Furthermore, the combined MD and NMR analysis revealed that fCS **10-c** possesses a well-defined extended conformation (Figure 3) not affected by significant conformational changes along the simulation. Moreover, Fuc units pointed out with respect to the GlcA-GalNAc skeleton and therefore the sulfate groups were well-ordered and regularly exposed on the glycan chain, creating a patch of solvent exposed negative charges, this suggesting the possibility of fCS **10-c** to form fibers.

## Conclusions

A semisynthetic access to structurally homogeneous LMW isomers of fCS polysaccharides carrying Fuc branches at a non-natural site, that is, position *O*-4 of GalNAc units, has been reported. The strategy relies upon the regioselective modification of the *E. coli* O5:K4:H4 sourced chondroitin as starting material to give a partially protected polysaccharide derivative carrying a single free hydroxyl per repeating unit, located at the GalNAc-4 site. This served as a polysaccharide acceptor in fucosylation reactions with differently protected Fuc donors. Completion of the semisyntheses, by orthogonal removal of temporary protecting groups, sulfation and global deprotection allowed the obtainment of three fCS isomers. Their structure was analyzed in full details by 2D-NMR spectroscopy, confirming the postulated features and demonstrating a rather high degree of structural homogeneity, at least comparable with that found in natural fCS polysaccharides and surely much higher with respect to semisynthetic fCSs accessed up to now. The conformational behavior was also investigated by NMR and molecular dynamics simulation. By comparison with data reported for a natural fCS, having the same sulfation pattern, but different branching site relative to Fuc decorations, it could be concluded that the semisynthetic fCS isomers having Fuc branches at GalNAc *O*-4 positions possess a different 3D arrangement, characterized by a much more extended conformation exposing the Fuc units, arranged almost perpendicular to GalNAc and GlcA residues. In light of these results, it will be very interesting to compare the bioactivity of natural fCSs with their semisynthetic isomers. Work is in progress to this aim and will be reported in due course. Moreover, the semisynthesis of further non-natural polysaccharides, carrying Fuc or even different branches at other and multiple positions of the GlcA-GalNAc backbone repeating unit, has also been planned for the near future in order to create a library of fCS isomeric structures to then be subjected to a comprehensive structure–activity relationships study. Last, but not least, we feel this work represents a significant step forward in the molecular complexity achievable through a glycosylation reaction. Indeed, its employment is very well established in carbohydrate chemistry,^54−56^ while its use for polysaccharide structural modification has been reported very seldom, in spite of its great potential for the obtainment of new polysaccharide-based biomaterials.

## Supporting Information Available

Experimental procedures, copies of 1D- and 2D-NMR spectra, complete chemical shift data assignments for the semisynthetic fCS polysaccharides, and figures of MD-derived conformers (PDF)
