# Correction to “Semisynthetic Isomers of Fucosylated Chondroitin Sulfate Polysaccharides with Fucosyl Branches at a Non-Natural Site”

**DOI:** 10.1021/acs.biomac.2c00045

**Published:** 2022-01-25

**Authors:** Giulia Vessella, Roberta Marchetti, Angela Del Prete, Serena Traboni, Alfonso Iadonisi, Chiara Schiraldi, Alba Silipo, Emiliano Bedini

The authors regret that the original version of this article unfortunately contained a mistake in all of the graphics depicting polysaccharide derivatives with fucosyl branches (Abstract Graphic, Scheme 1, Scheme 3, and Chart 1). In particular, a d-fucose unit was erroneously drawn instead of the l residue. The corrected graphics are given here. The authors would like to apologize for any inconvenience caused.

Abstract Graphic:

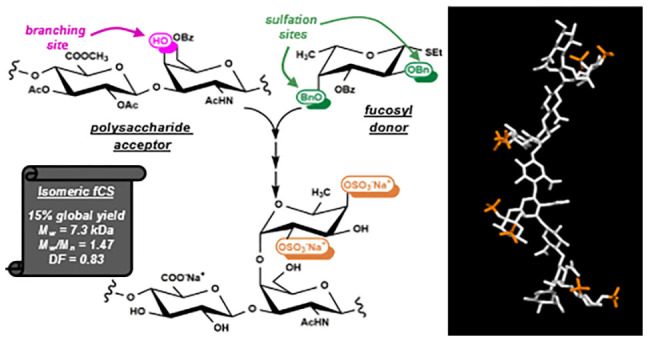



**Scheme 1:**
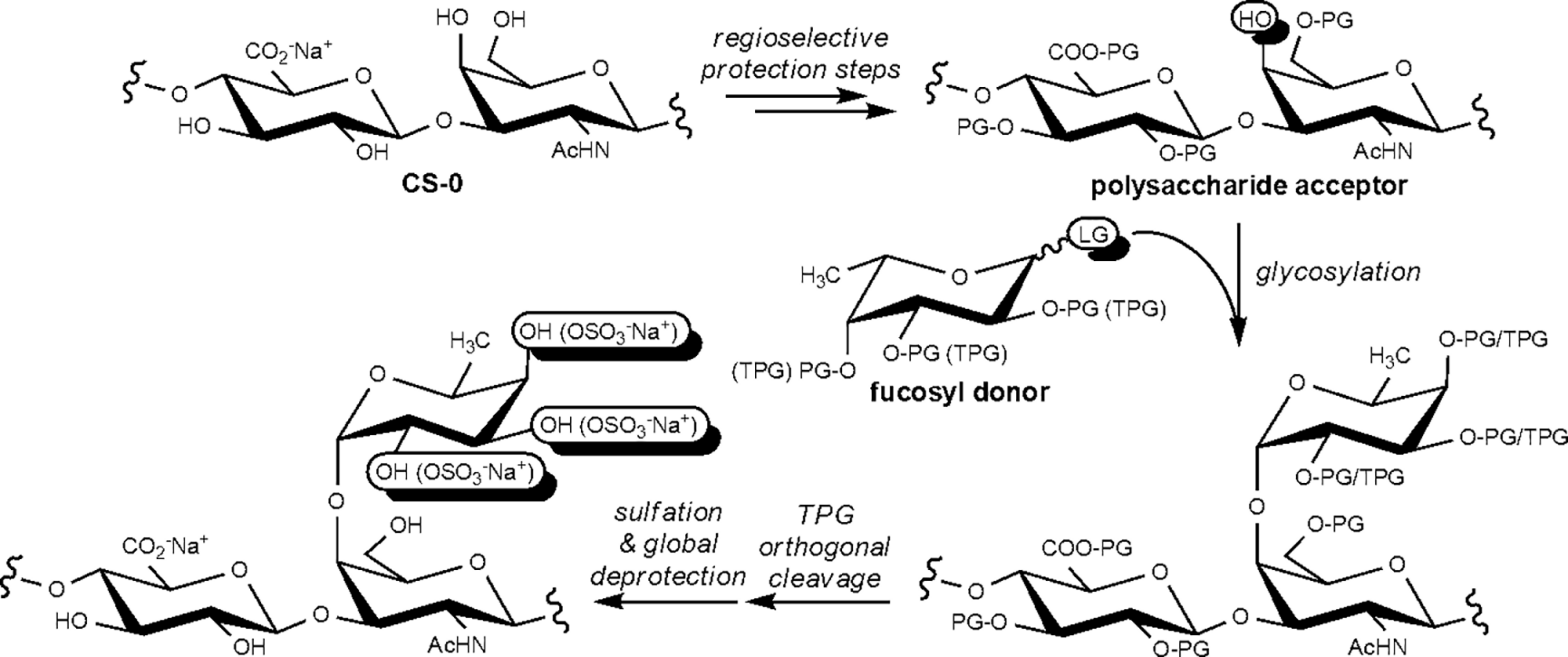
General Strategy to Access Semisynthetic Isomers of fCS Polysaccharides from Microbial-Sourced Unsulfated Chondroitin

**Scheme 3:**
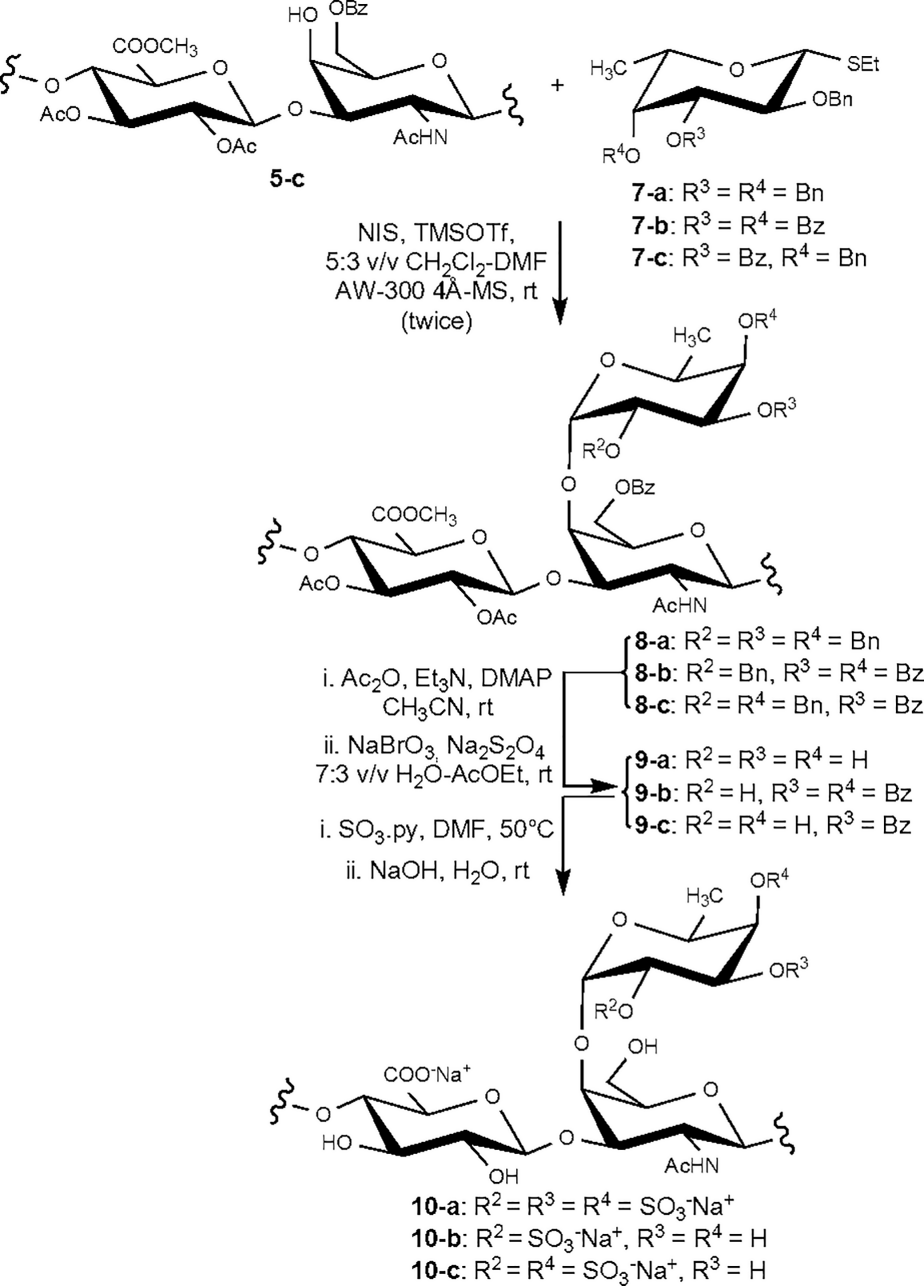
Semisynthesis of fCS Polysaccharides **10-a–c**

**Chart 1:**
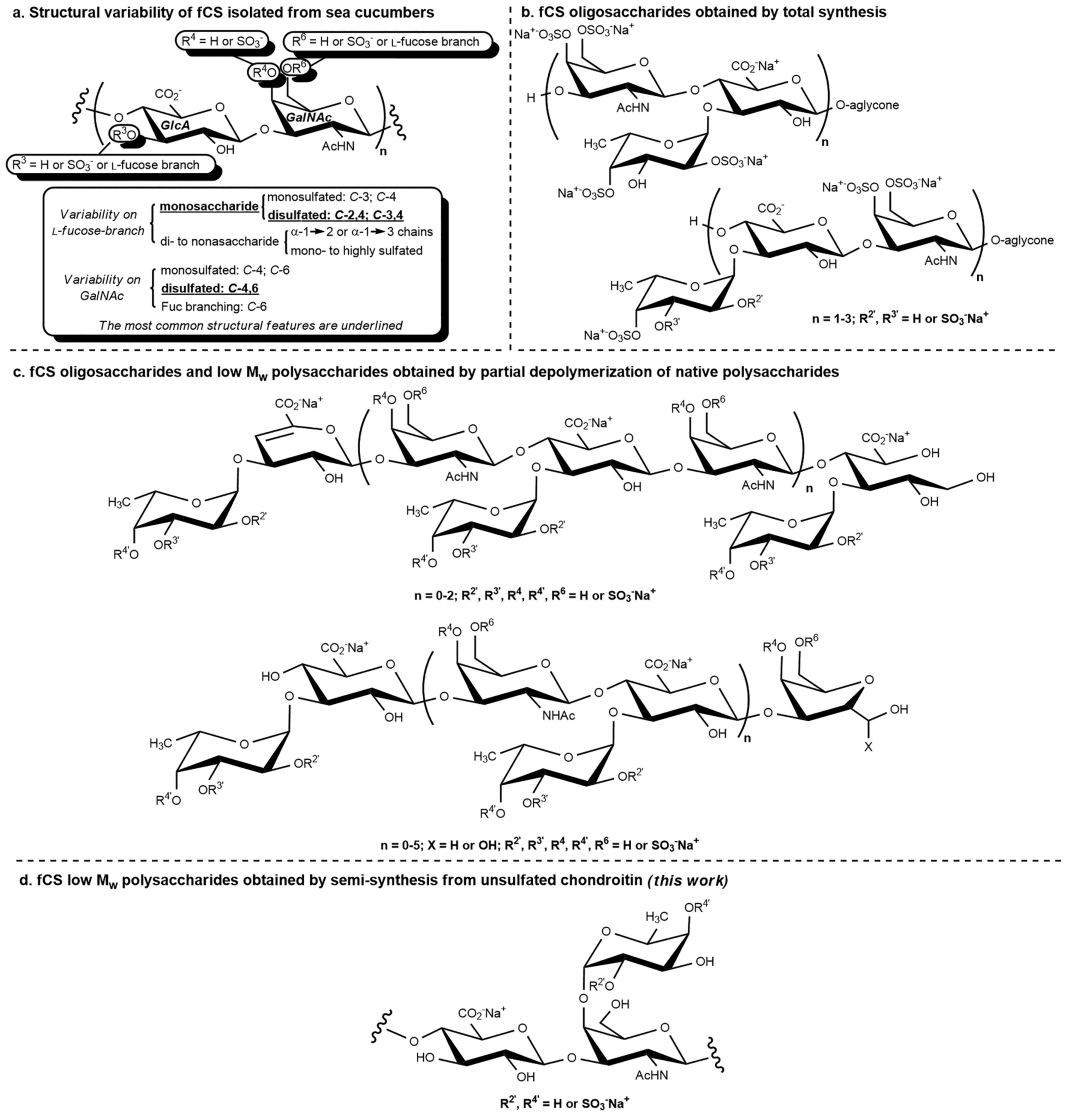
Structure of Natural fCS Polysaccharides and Chemically Obtained Oligosaccharides and Low *M*_w_ Polysaccharides

