# Supplementary material for: Semisynthetic Isomers of Fucosylated Chondroitin Sulfate Polysaccharides with Fucosyl Branches at a Non-Natural Site
Source: Biomacromolecules. 2021 Nov 14;22(12):5151–61. doi: 10.1021/acs.biomac.1c01112 (PMC8672353; doi:10.1021/acs.biomac.1c01112)
Supplement: Supplementary file 1 — bm1c01112_si_001.pdf [file bm1c01112_si_001.pdf]

Supporting Information

SEMI-SYNTHETIC ISOMERS OF FUCOSYLATED CHONDROITIN SULFATE POLYSACCHARIDES WITH FUCOSYL BRANCHES AT A NON-NATURAL SITE

GIULIA VESSELLA,[1] ROBERTA MARCHETTI,[1] ANGELA DEL PRETE,[1,2] SERENA TRABONI,[1]
ALFONSO IADONISI,[1] CHIARA SCHIRALDI,[3] ALBA SILIPO,[1] EMILIANO BEDINI,[1]

[1]Department of Chemical Sciences, University of Naples Federico II,
Complesso Universitario Monte S.Angelo, via Cintia 4, I-80126 Napoli, Italy
[2]Department of Chemistry, University of Natural Resources and Life Sciences,
Muthgasse 18, 1190 Vienna, Austria
[3]Department of Experimental Medicine, Section of Biotechnology,
University of Campania "Luigi Vanvitelli", via de Crecchio 7, I-80138 Napoli, Italy

Table of Contents

Table S1: ^{1}H and ^{13}C chemical shift assignments for **10-a,c** S-3

Figure S1: ^{1}H NMR spectrum of **2** S-4

Figure S2: ^{1}H NMR spectrum of **3** S-4

Figure S3: ^{1}H NMR spectrum of **4** S-5

Figure S4: ^{1}H NMR spectrum of **5-a** S-5

Figure S5: ^{1}H NMR spectrum of **5-f** S-6

Figure S6: ^{1}H and $^{1}H,^{13}C$-DEPT-HSQC NMR spectra of **6-a** S-6

Figure S7: ^{1}H and $^{1}H,^{13}C$-DEPT-HSQC NMR spectra of **6-b** S-7

Figure S8: ^{1}H and $^{1}H,^{13}C$-DEPT-HSQC NMR spectra of **6-c** S-7

Figure S9: ^{1}H and $^{1}H,^{13}C$-DEPT-HSQC NMR spectra of **6-d** S-8

Figure S10: ^{1}H and $^{1}H,^{13}C$-DEPT-HSQC NMR spectra of **6-e** S-8

Figure S11: ^{1}H and $^{1}H,^{13}C$-DEPT-HSQC NMR spectra of **6-f** S-9

Figure S12: ^{1}H NMR spectrum of **9-a** S-9

Figure S13: ^{1}H DOSY NMR spectrum of **10-c** S-10

Figure S14: ^{1}H and NOESY NMR spectra of **10-c** S-10

Figure S15: ^{1}H and $^{1}H,^{13}C$-DEPT-HSQC, COSY, TOCSY and NOESY NMR spectra of **10-a** S-11

Figure S16: ^{1}H and $^{1}H,^{13}C$-DEPT-HSQC, COSY, TOCSY and NOESY NMR spectra of **10-b** S-11

Figure S17: ^{1}H and $^{1}H,^{13}C$-DEPT-HSQC, COSY, TOCSY and NOESY NMR spectra of **10-c** S-12

Figure S18 Adiabatic energy maps for the basic constituent disaccharides of fCS-**10c** repeating unit S-12

Figure S19: Scatter plots of Φ *vs* Ψ along the MD simulation for the central disaccharide units contained in fCS oligosaccharide S-13

Figure S20: Representative fCS-**10c** conformers, and polar inter-residue interactions stabilizing the overall fCS structure S-14

Table S1: ^{1}H (plain) and ^{13}C (italic) chemical shift assignments for target polysaccharides **10-a,c**[a,b]

		**1**	**2**	**3**	**4**	**5**	**6**	*Other signals*
**10-a**	*GalNAc^{Fuc}*	4.56<br>*102.2*	4.13<br>*52.7*	3.94<br>*81.4*	4.20<br>*76.7*	3.76<br>*76.6*	3.68-3.82<br>*62.5*	NAc<br>2.00-2.06<br>23.8
	GalNAc^{noFuc}	4.50<br>*102.2*	3.99<br>*52.5*	3.78<br>*81.5*	4.10<br>*68.7*	3.69<br>*76.3*		
	GlcA	4.46<br>*105.8*	3.34<br>*73.7*	3.57<br>*75.1*	3.75<br>*79.7*	3.68<br>*78.0*	---	---
	α-Fuc-(1→4)-GalNAc	5.38<br>*100.5*	3.76<br>*69.8*	3.92<br>*70.9*	3.81<br>*73.1*	4.08<br>*68.7*	1.18-1.27<br>*16.6*	---
**10-b**	*GalNAc^{Fuc}*	4.52-4.59<br>*102.4*	3.98–4.12<br>*52.5-52.8*	n.d.[c]	4.22<br>*74.6*	3.78<br>*76.8*	3.71-3.83<br>*62.4*	NAc<br>1.99-2.06<br>*23.7*
	GalNAc^{noFuc}			3.79<br>*81.4*	4.01<br>*68.6*	n.d.[c]		
	GlcA^{i}	4.48<br>*105.6*	3.36<br>*73.8*	3.58<br>*75.1*	3.75<br>*81.5*	3.67<br>*77.9*	---	---
	GlcA^{ii}	4.38<br>*106.1*	3.53<br>*73.6*	3.64<br>*77.9*	n.d.[c]	n.d.[c]		
	α-Fuc2S-(1→4)-GalNAc	5.67<br>*98.2*	4.42<br>*76.3*	4.10<br>*68.8*	3.90<br>*73.2*	4.05<br>*68.5*	1.19-1.26<br>*16.4*	---
**10-c**	*GalNAc^{Fuc}*	4.61<br>*102.9*	4.07<br>*53.2*	4.03<br>*80.6*	4.26<br>*74.5*	3.75<br>*76.8*	3.66-3.84<br>*62.4*	NAc<br>1.94-2.05<br>*23.8*
	GalNAc^{noFuc}	4.54<br>*102.6*	3.99<br>*52.5*	3.80<br>*81.8*	n.d.[c]			
	GlcA^{i}	4.49<br>*105.7*	3.35<br>*73.6*	3.61<br>*74.9*	3.76<br>*81.7*	3.79<br>*76.7*	---	---
	GlcA^{ii}	4.42<br>*106.0*	3.50<br>*73.3*					
	α-Fuc2,4S-(1→4)-GalNAc	5.71<br>*98.0*	4.43<br>*76.4*	4.19<br>*67.8*	4.69<br>*82.1*	4.06<br>*68.0*	1.21-1.36<br>*17.0*	---

[a] Chemical shifts are referred to residues in italic characters and are expressed in δ relative to internal acetone (^{1}H: $(CH_3)_2CO$ at δ = 2.22 ppm; ^{13}C: $(CH_3)_2CO$ at δ = 31.5 ppm).
[b] The superscripts *i* and *ii* to residue names represent the same residue type with different neighbouring residues.
[c] Not detectable.

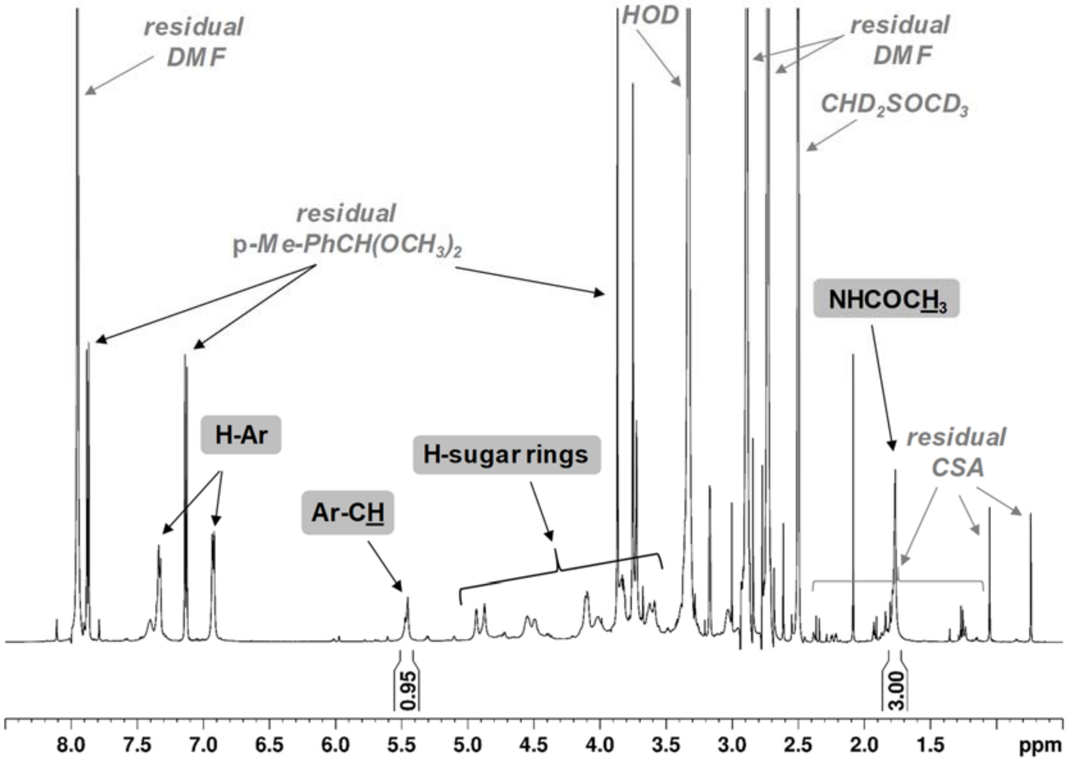

Figure S1: ^{1}H NMR spectrum (600 MHz, DMSO-d_6, 298 K) of **2**

(polysaccharide signal assignments are enclosed in rectangles)

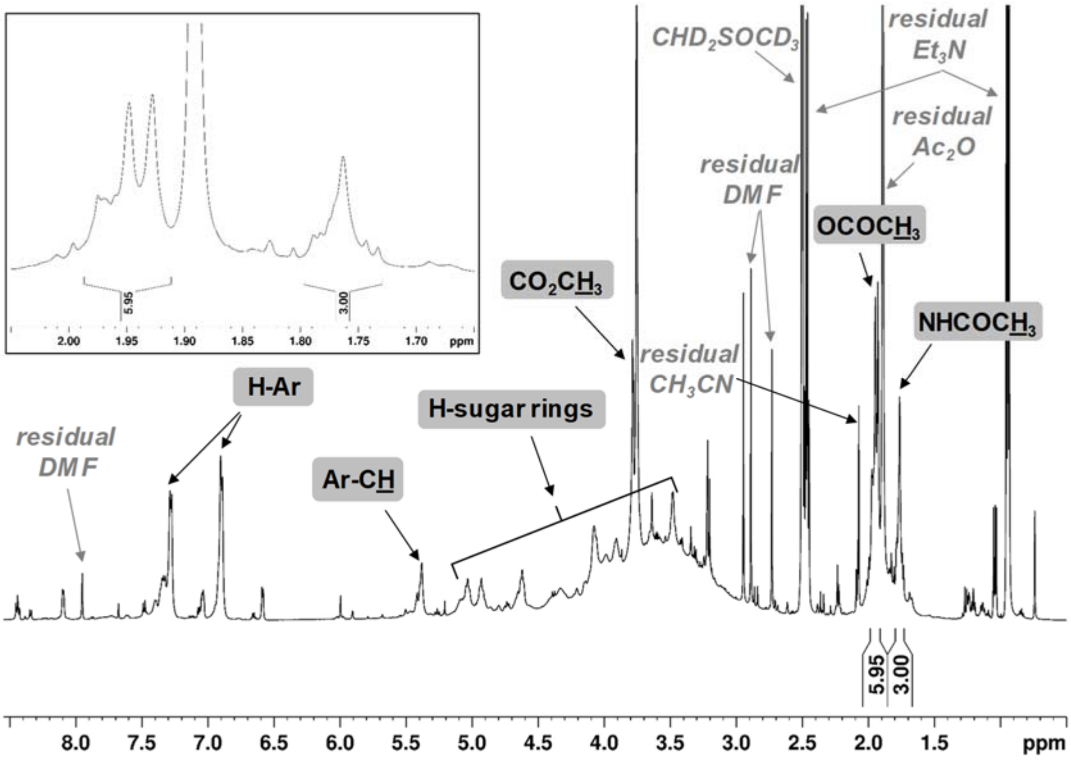

Figure S2: ^{1}H NMR spectrum (600 MHz, DMSO-d_6, 298 K) of **3**

(polysaccharide signal assignments are enclosed in rectangles)

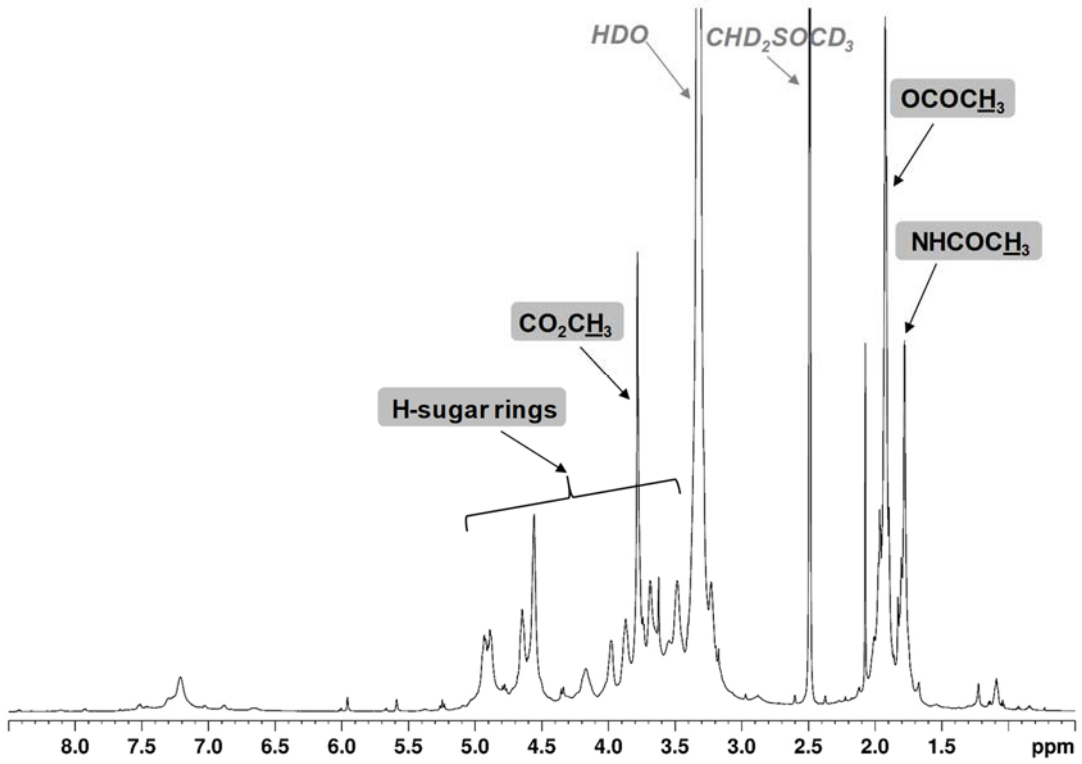

Figure S3: ^{1}H NMR spectrum (600 MHz, DMSO-d_6, 298 K) of **4**

(polysaccharide signal assignments are enclosed in rectangles)

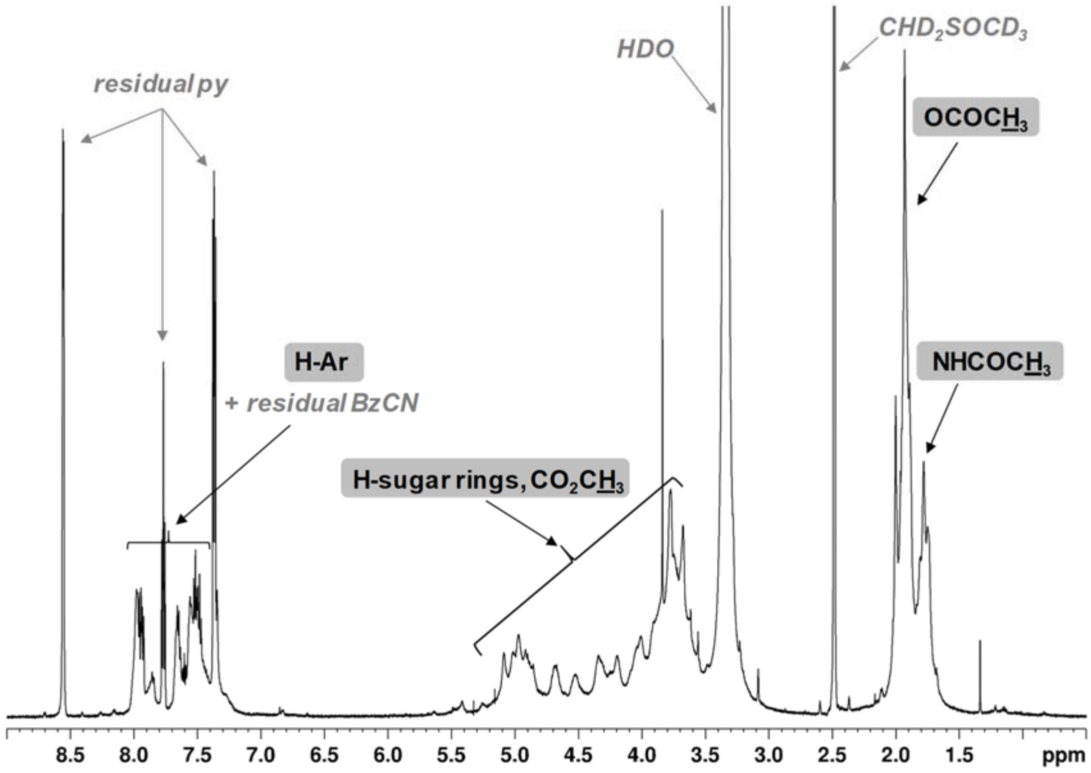

Figure S4: ^{1}H NMR spectrum (600 MHz, DMSO-d_6, 298 K) of **5-a**

(polysaccharide signal assignments are enclosed in rectangles)

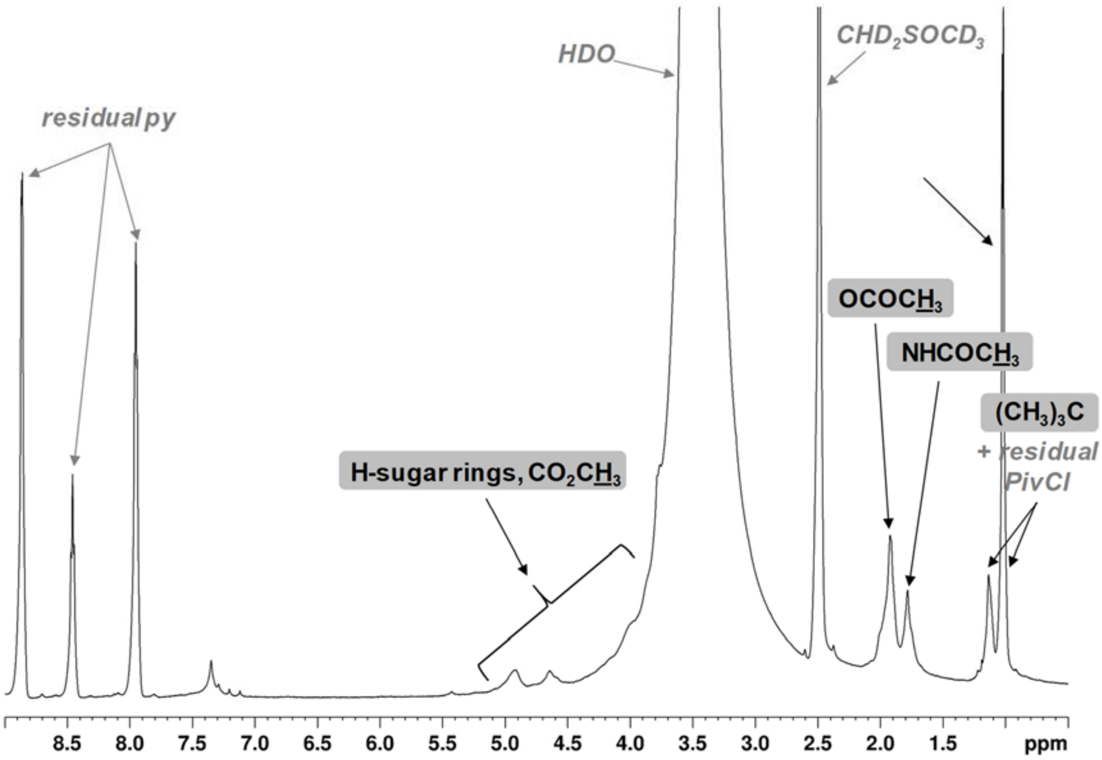

Figure S5: ^{1}H NMR spectrum (600 MHz, DMSO-d_6, 298 K) of **5-f**

(polysaccharide signal assignments are enclosed in rectangles)

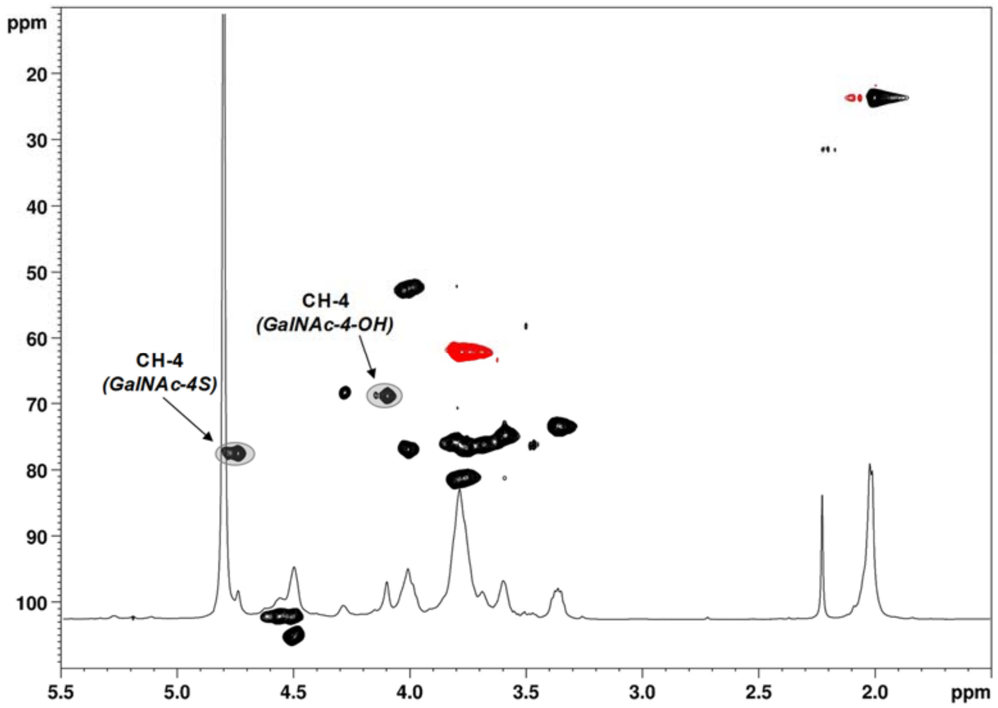

Figure S6: ^{1}H and $^{1}H,^{13}C$-DEPT-HSQC NMR spectra (600 MHz, 298K, D_2O) of **6-a** (densities enclosed in the highlighted areas were integrated for estimation of relative amounts of differently sulfated GalNAc units)

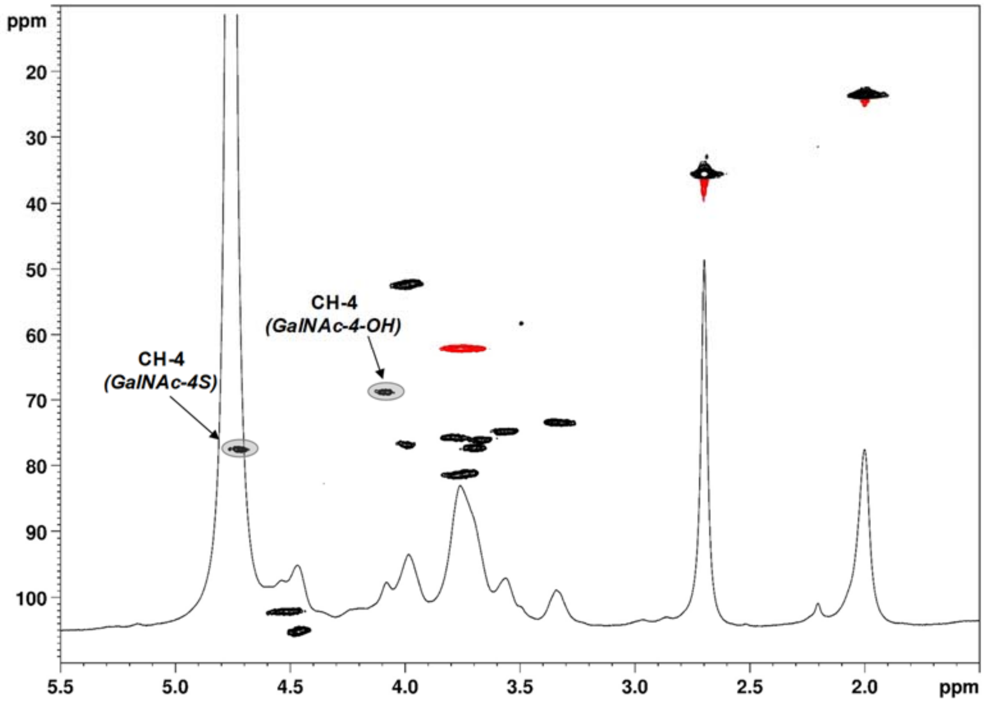

Figure S7: ^{1}H and $^{1}H,^{13}C$-DEPT-HSQC NMR spectra (400 MHz, 298K, D_2O) of **6-b** (densities enclosed in the highlighted areas were integrated for estimation of relative amounts of differently sulfated GalNAc units)

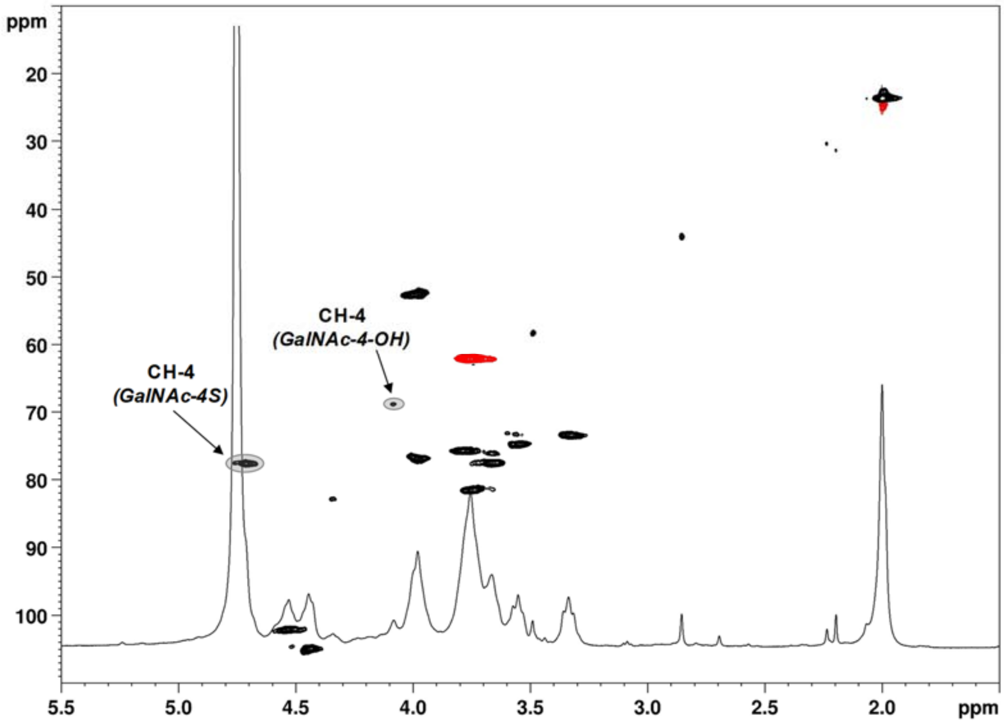

Figure S8: ^{1}H and $^{1}H,^{13}C$-DEPT-HSQC NMR spectra (400 MHz, 298K, D_2O) of **6-c** (densities enclosed in the highlighted areas were integrated for estimation of relative amounts of differently sulfated GalNAc units)

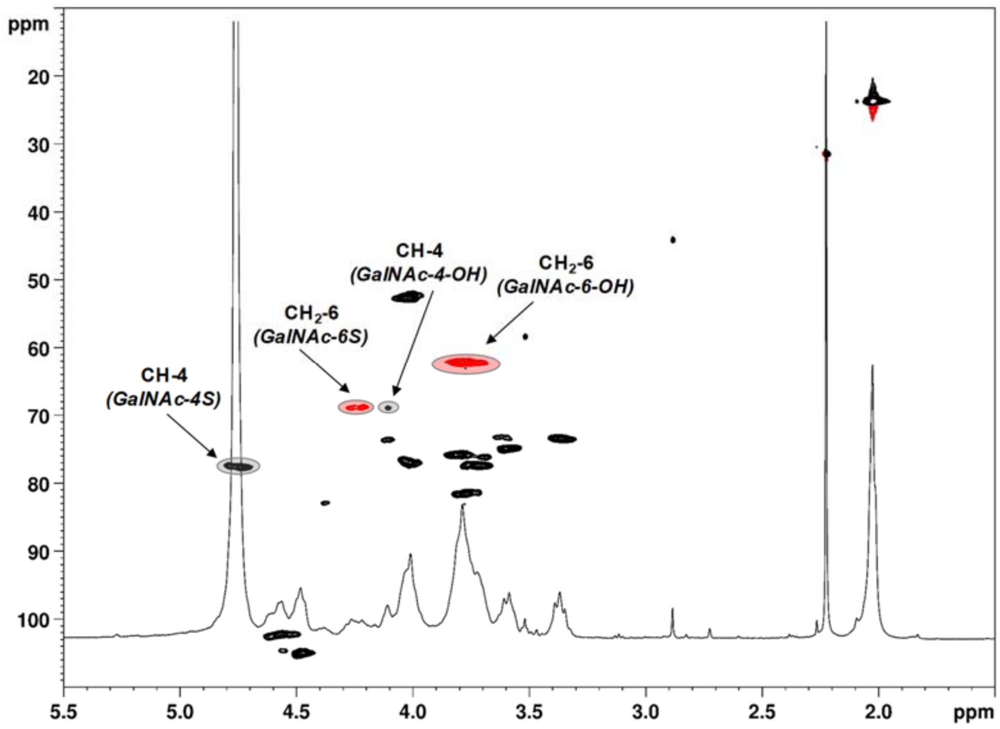

Figure S9: ^{1}H and $^{1}H,^{13}C$-DEPT-HSQC NMR spectra (400 MHz, 298K, D_2O) of **6-d** (densities enclosed in the highlighted areas were integrated for estimation of relative amounts of differently sulfated GalNAc units)

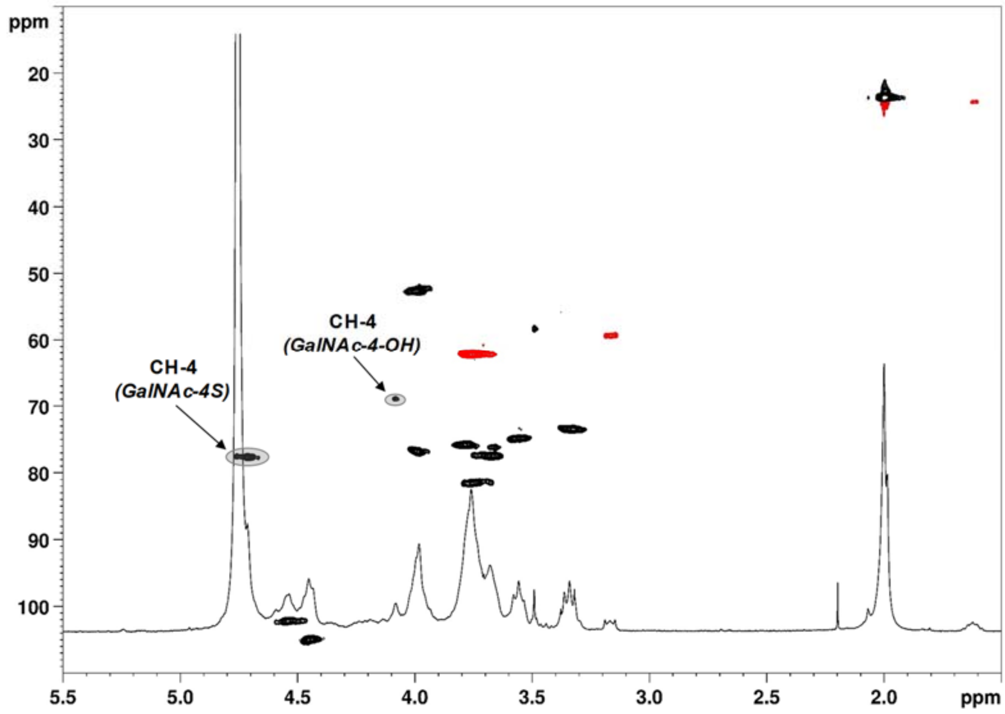

Figure S10: ^{1}H and $^{1}H,^{13}C$-DEPT-HSQC NMR spectra (400 MHz, 298K, D_2O) of **6-e** (densities enclosed in the highlighted areas were integrated for estimation of relative amounts of differently sulfated GalNAc units)

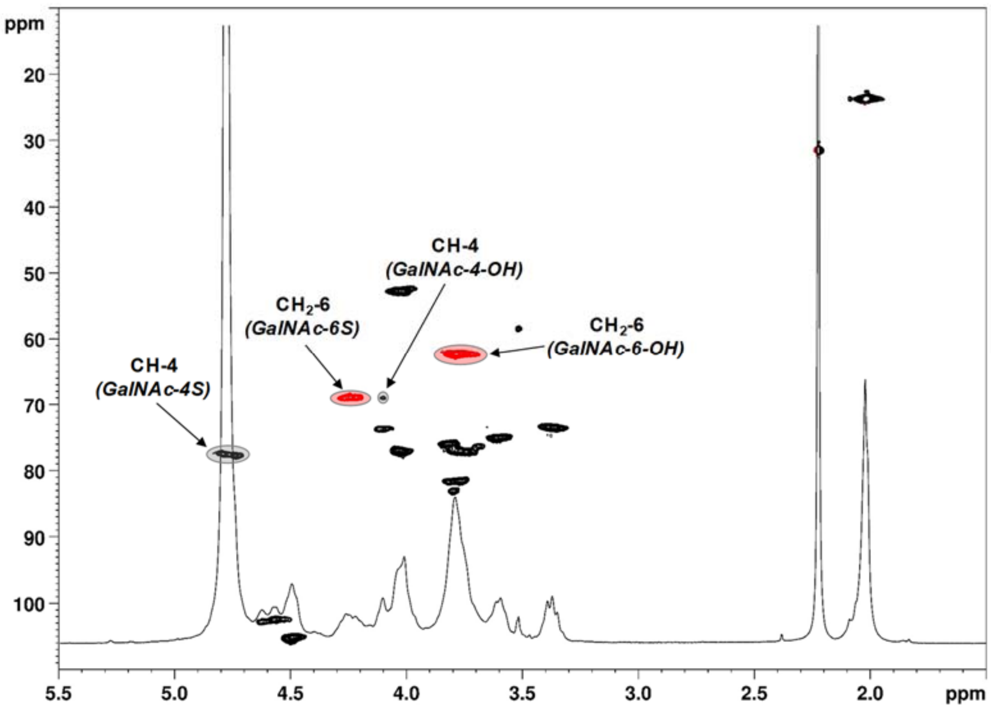

Figure S11: ^{1}H and ^{1}H,^{13}C-DEPT-HSQC NMR spectra (400 MHz, 298K, D_2O) of **6-f** (densities enclosed in the highlighted areas were integrated for estimation of relative amounts of differently sulfated GalNAc units)

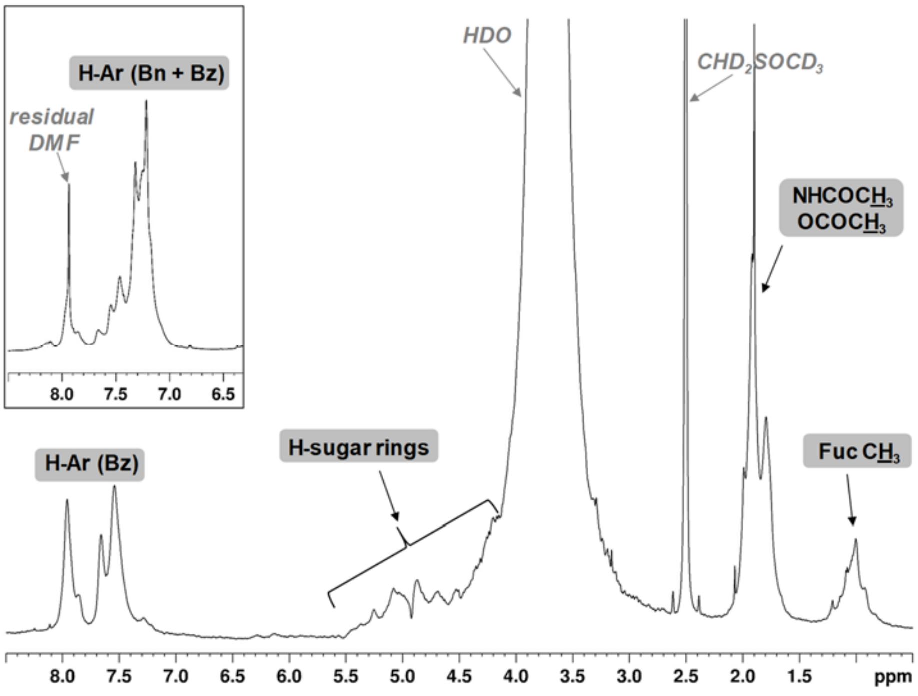

Figure S12: ^{1}H NMR spectrum (600 MHz, DMSO-d_6, 298 K) of **9-a**

(zoom of ^{1}H NMR spectrum of precursor **8-a** in the upper left box;

polysaccharide signal assignments are enclosed in rectangles)

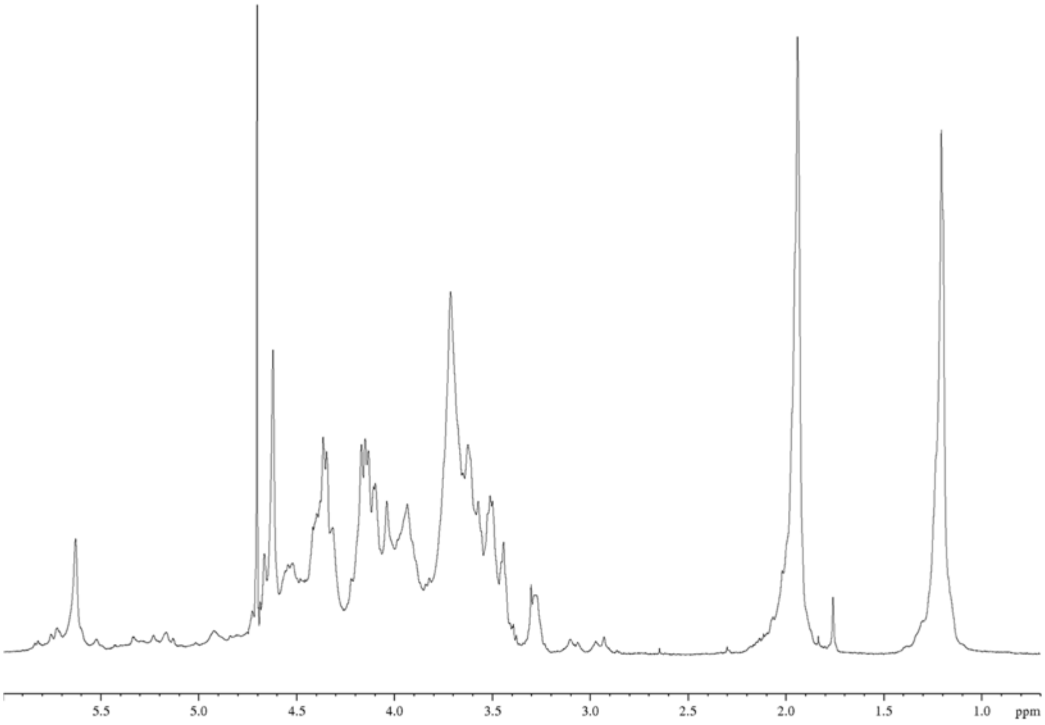

Figure S13: ^{1}H DOSY NMR spectrum (600 MHz, D_2O, 298 K) of **10-c**

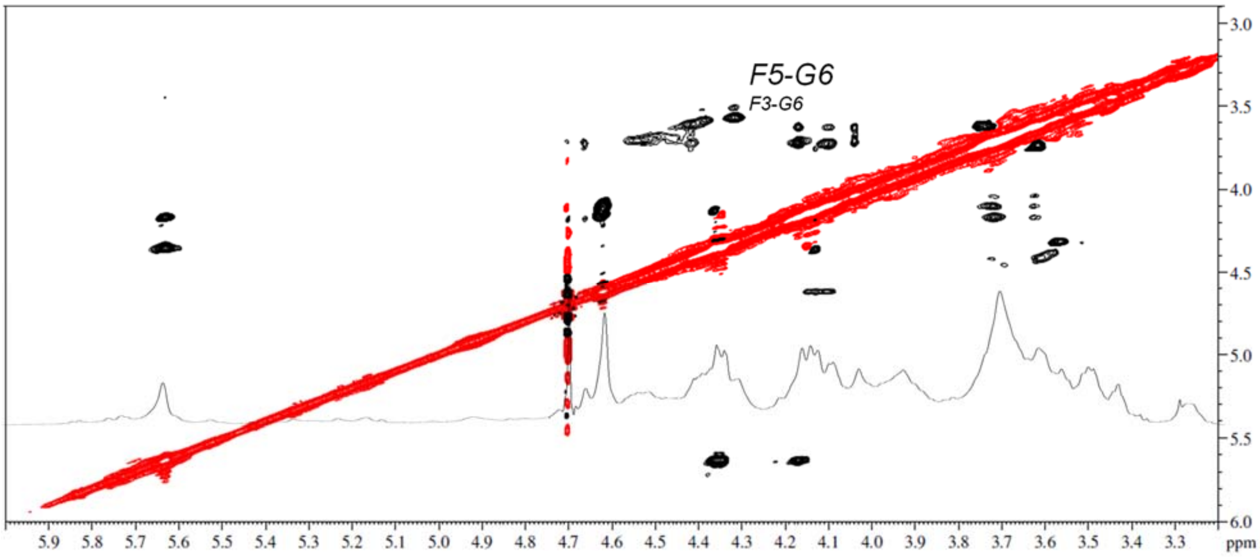

Figure S14: ^{1}H and NOESY NMR spectra (600 MHz, D_2O, 298 K) of **10-c (**only some of the assignments are indicated)

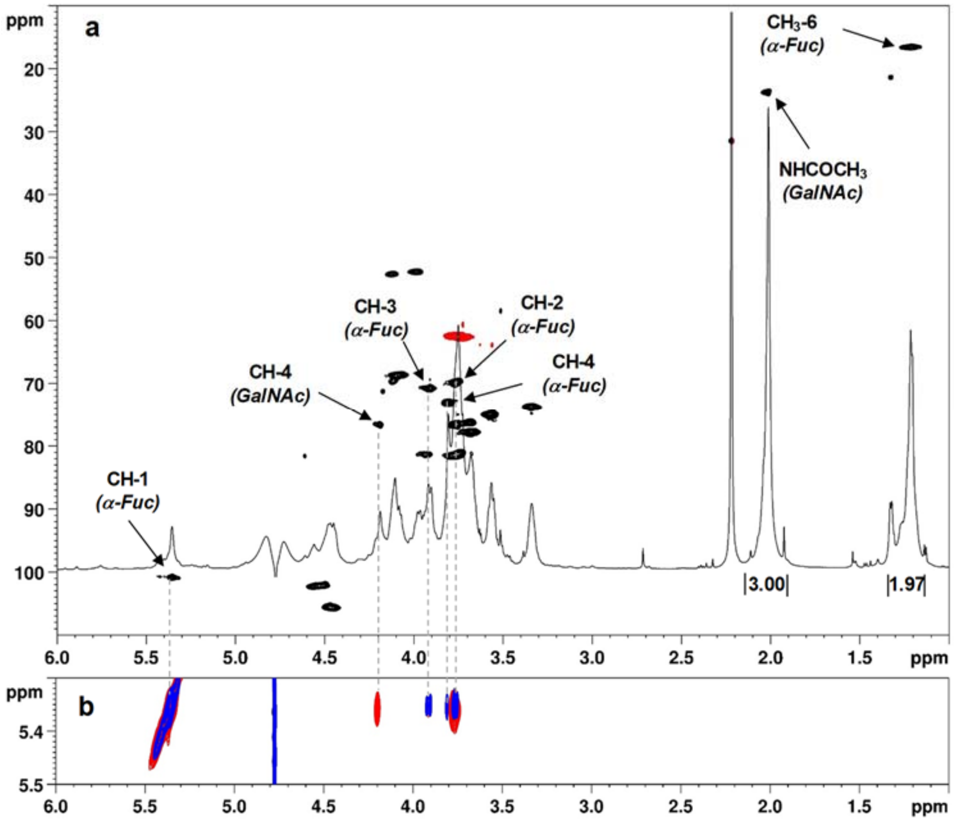

Figure S15: (a) ^{1}H and ^{1}H,^{13}C-DEPT-HSQC and (b) COSY (black), TOCSY (blue) and NOESY (red) NMR spectra (600 MHz, D_2O, 298 K, zoom) of **10-a (**only some of the assignments are indicated, for full assignments see TableS1)

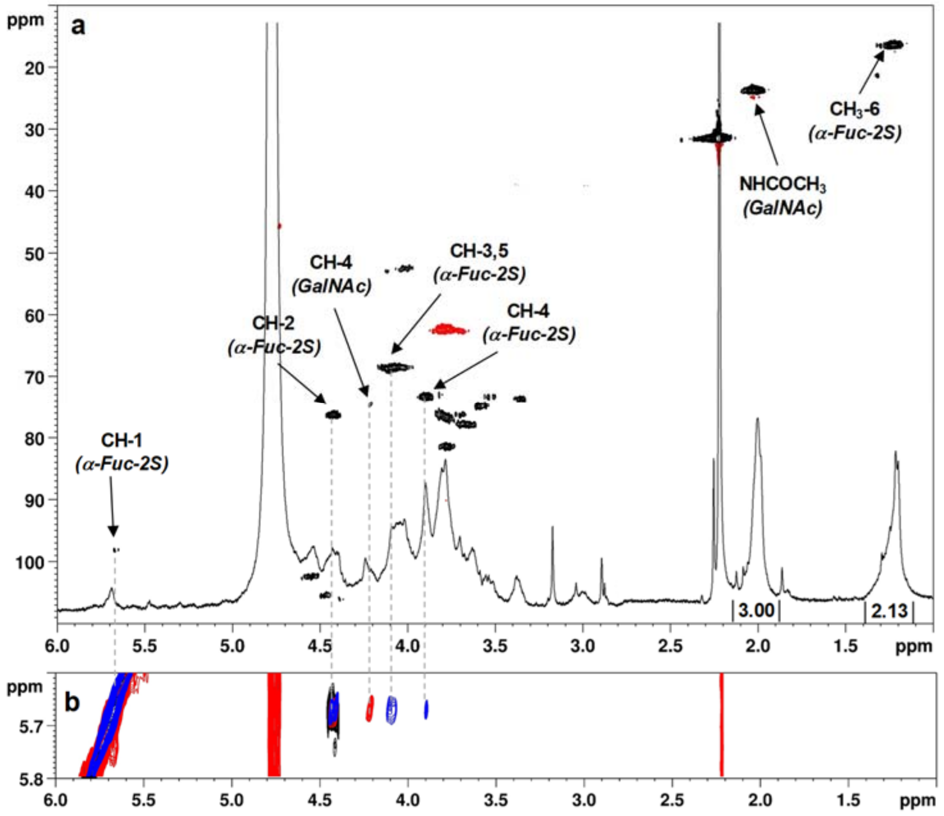

Figure S16: (a) ^{1}H and ^{1}H,^{13}C-DEPT-HSQC and (b) COSY (black), TOCSY (blue) and NOESY (red) NMR spectra (600 MHz, D_2O, 298 K, zoom) of **10-b (**only some of the assignments are indicated, for full assignments see TableS1)

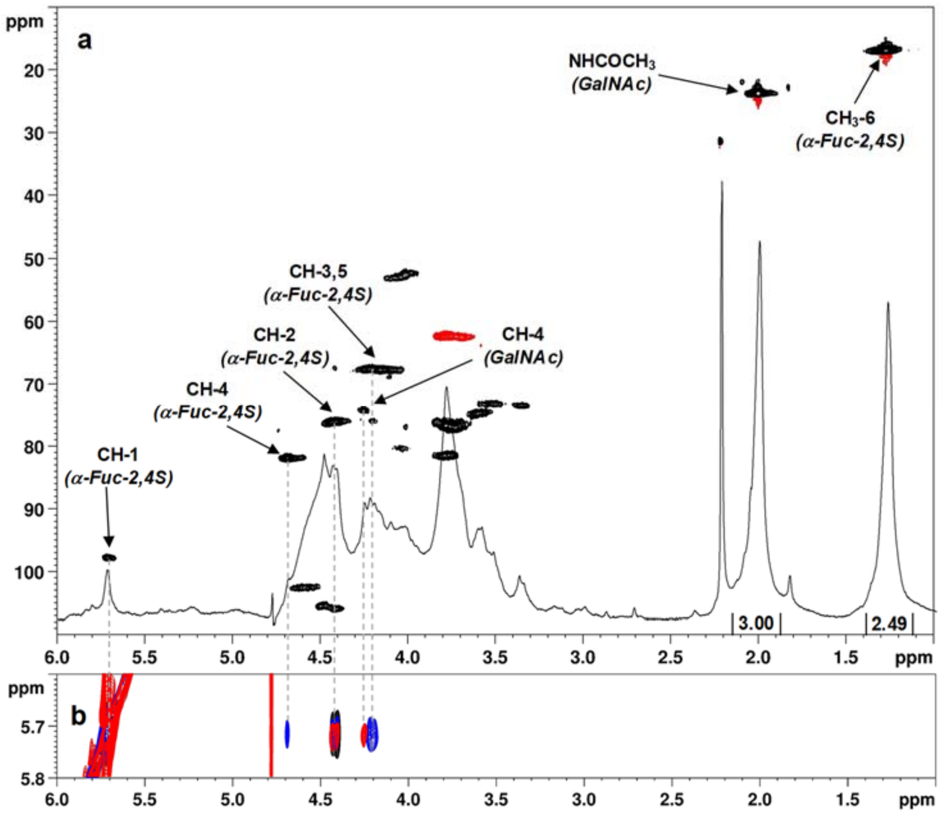

Figure S17: (a) ^{1}H and $^{1}H,^{13}C$-DEPT-HSQC and (b) COSY (black), TOCSY (blue) and NOESY (red) NMR spectra (600 MHz, D_2O, 298 K, zoom) of **10-c (**only some of the assignments are indicated, for full assignments see TableS1)

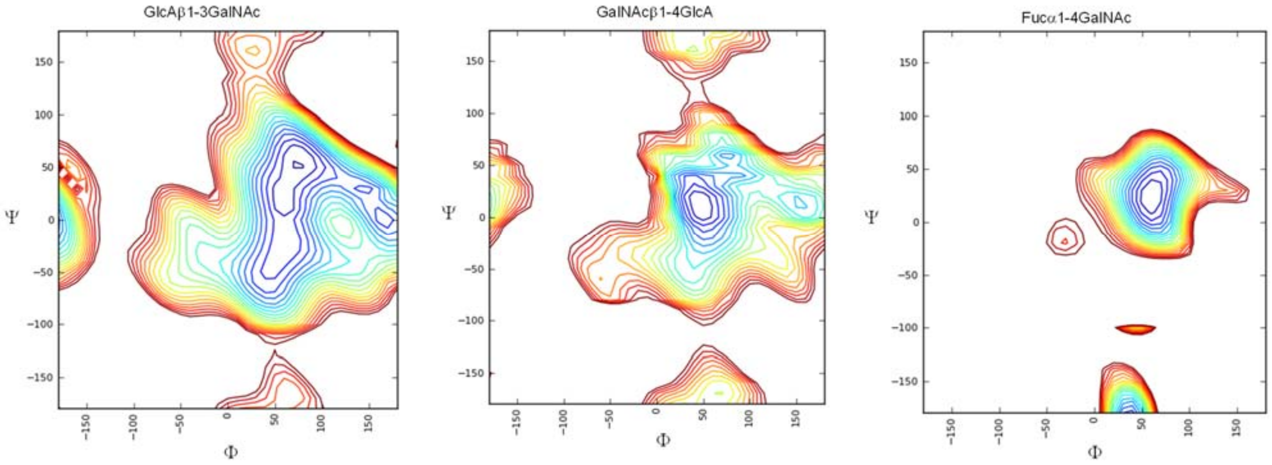

Figure S18: Adiabatic energy maps for the basic constituent disaccharides of fCS **10c** repeating unit. The glycosidic torsions were defined as follows: Φ (H1-C1-O-CX') and Ψ (C1-O-CX'-HX').

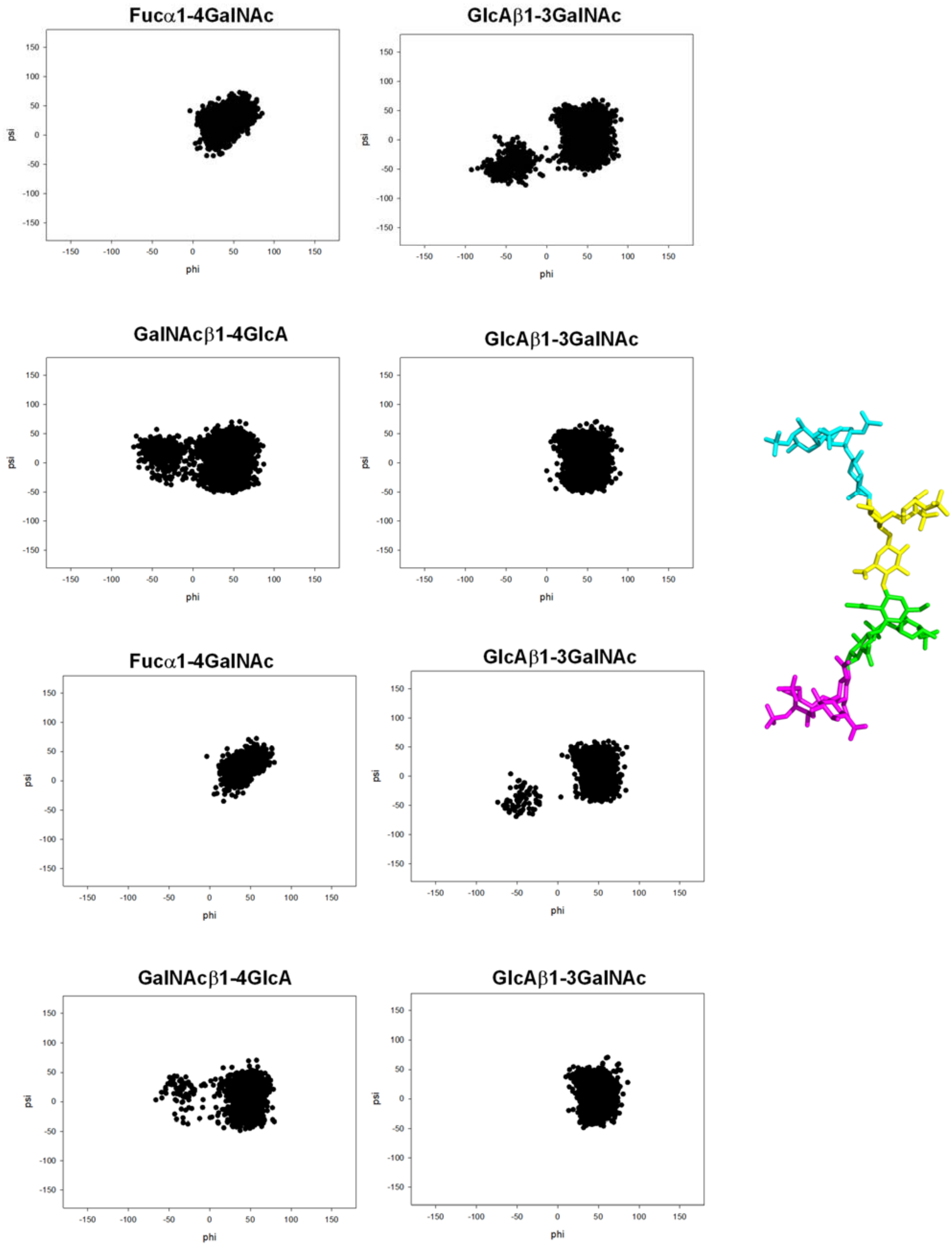

Figure S19: Scatter plots of Φ *vs* Ψ along the MD simulation for the central disaccharide units contained in fCS oligosaccharide

a

b

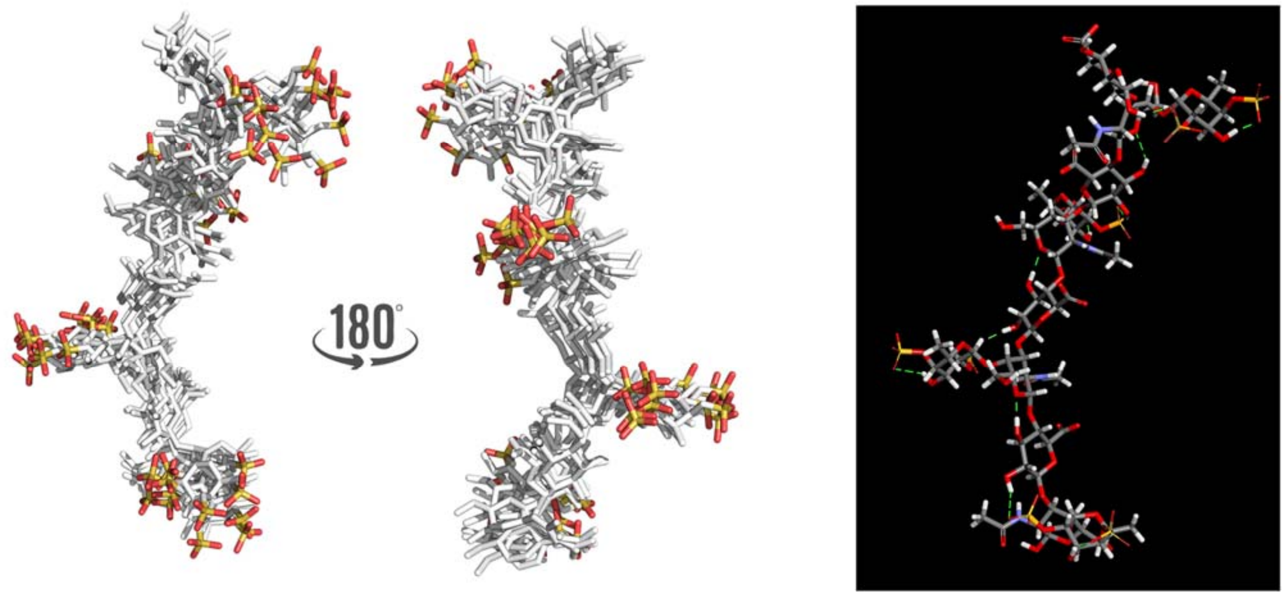

Figure S20: a) Representative fCS **10c** conformers (sugar skeleton white colored, sulfate groups red-orange colored). b) Polar inter-residue interactions (green dashed lines) stabilizing the overall fCS structure
